# Evaluation of improved cassava genotypes for fresh root yield and yield components in demand creation trial

**DOI:** 10.3389/fpls.2025.1564393

**Published:** 2025-06-06

**Authors:** T.C. Onyenali, T.O. Ogwuche, J. Onyeka, E.M. Diebiru-Ojo, I. Ikechukwu, S.P. Abah, I. Okwuonu, E.A. Ameh, L. Sanni, A.P. Kulakow, C.N. Egesi

**Affiliations:** ^1^ Biotechnology Department, National Crops Research Institute, Umuahia, Abia State, Nigeria; ^2^ International Institute of Tropical Agriculture, Ibadan, Nigeria; ^3^ Sahel Consulting Agriculture and Nutrition Limited, Abuja, Nigeria

**Keywords:** demand creation trial, cassava storage roots, genotype x environment interaction, estimated marginal means, commercial processors demand creation trial, cassava, geneotype x environment interactionGEI, commercial processors

## Abstract

Fresh yield and dry matter concentration are important traits that promote the adoption of improved cassava varieties by processors and their out-grower farmers in southeastern Nigeria. Therefore, for wide adoption of improved cassava varieties developed by the International Institute of Tropical Agriculture, Ibadan, and National Root Crops Research Institute, Umudike, there is a need to evaluate improved varieties for these attributes continuously, hence the need to explore variety decision support tool like the demand creation trials (DCT) to evaluate cassava varieties with farmers and processors utilizing the best agronomic practices. The DCTs were conducted in three environments during the 2020–2021 and 2021–2022 cropping seasons with two replications. A randomised complete block design (RCBD) was used for this study with four improved varieties. The plot size was 320 m2 with a 1 m x 0.8 m spacing. The traits evaluated were Plant Vigor (PV), number of storage roots, fresh yield, dry yield, Dry Matter Content (DMC), and bundle estimation. The traits were subjected to a GGE biplot in R software to identify high-yielding and stable genotypes. The estimated marginal means (EMM) for cassava yieldwere compared across different clones, environments, and years, revealing significant differences (p < 0.01). According to the result, Variety TMS13F1160P0004 had the highest fresh root yields of 37.61 t/ha and 35.46 t/ha in 2021 at the Abia and Owerri locations, respectively. Additionally, variety TMS13F1160P0004 had the best dry yield in 2021, with 12.81 t/ha in Abia and 11.73 t/ha in Owerri. According to the genotype by environment interaction analysis, yield estimates varied greatly, especially for CR36-5 and IBA980693, although varieties TMS13F1160P0004 and IBA961632 were more stable across environments. For dry matter content (%), there was no discernible difference across the varieties. These findings showed the significance of conducting DCTs across various environmentss with independent cassava processors to to guide their choices of cassava varieties for factory operations.

## 1 Introduction

*Manihot esculenta* Crantz, commonly known as Cassava, is a tropical root crop native to South America, primarily cultivated for its carbohydrate-rich storage roots. Presently, cassava is extensively grown across tropical regions of South America, Africa, and Asia, serving as a dietary staple for approximately 800 million people globally. In sub-Saharan Africa, cassava plays a pivotal role in ensuring food security. Beyond human nutrition, cassava starch has applications in various industries, including pharmaceuticals, textiles, paper production, and biofuel (Nassar and Ortiz, 2006: Lin et al., 2019).

Nigeria is the largest producer of cassava globally, contributing approximately 21% of the world’s output (FAO, 2023). Despite its pivotal role, the yield potential of cassava has not been fully realized due to various factors such as pest and disease pressure (Lin et al., 2019; McCallum et al., 2017), sub-optimal agronomic practices (Hershey, 1988), and poor adoption of improved varieties despite its availability (Mwebaze et al., 2024). Improved cassava varieties have been developed to address these challenges, with enhanced disease resistance, higher yields, and better agronomic traits than local varieties. These improved varieties offer significant advantages to smallholder farmers, potentially enhancing productivity and profitability. According to Acheampong et al (2022) *, adopting improved varieties in Ghana significantly increased* cassava yields, with adopters experiencing up to an 18 t/ha increase. However, despite the development of improved varieties resistant to cassava mosaic disease, bacterial blight, and green mites, adoption remains low (Hahn et al., 1979; Mwebaze et al., 2024). To enhance productivity and boost adoption, targeted extension approaches, awareness creation, and strategies to promote existing improved varieties are recommended (Mwebaze et al., 2024; Acheampong et al., 2022).

Demand creation trials are designed to bridge this gap (Diagne, 2009) by demonstrating the benefits of these varieties under real-world conditions, showcasing their superior traits to farmers and stakeholders, and thus facilitating wider adoption (Diagne, 2009). Southeast Nigeria, a major cassava-growing region (Udensi et al., 2011), provides a unique context for evaluating improved cassava varieties, given its diverse agroecological zones and the prominence of cassava-based industries. Many cassava root farmers have benefitted little in selling roots to industries and processors because they do not cultivate the required varieties. When buying roots, processors look out for traits such as starch and flour content in addition to fresh root yield based on feedback from the demand creation trials (Ogwuche et al., 2023). The demand for cassava is rising, particularly with more industries utilizing roots as raw materials (Ogwuche et al., 2023).

Stability refers to performance with changing environmental factors over time within a given location (Yan, 2001). Stability is highly relevant for plant breeders developing genotypes adapted to various environmental conditions (Mühleisen et al., 2014). Yield stability has a national and global dimension in the context of food security (Kalkuhl et al., 2016). Large variations of yield from year to year or from location to location are problematic as times of dearth and hunger cannot always and fully be compensated by higher yields (Abbo et al., 2010), thereby leading to potential conflicts over resources.

Genotype x environment interaction (GEI) refers to the variation in the response of genotypes to varied environmental (season, location, years, crop management practices, etc) conditions such that genotypes with desirable performance under a growing condition may be poor in another environment (Ceccarelli, 1996). However, understanding GEI patterns from GEI analyses has contributed tremendously to cultivar development. For instance, GEI has necessitated multi-environment trials (METS) from which breeders have identified genotypes that are adapted to a particular environment and those superior across several growing regions (Annicchiarico and Mariani, 1996; Kvitschal et al., 2006). Test environments have also been categorized and representative sites identified, eliminating unnecessary test locations and reducing the cost of cultivar evaluation (Yan, 2001). The direct implication of a significant GEI is the need to select genotypes based on consistent positive performance over a wide range of environments. Selecting high-yielding but unstable genotypes in a breeding program or commercial farm will lead to devastating results.

Plant breeders have thus developed several statistical techniques, including the Genotype Genotype x environment (GGE) biplot by Yan (2001), to aid decisions on the superiority or desirability of genotypes and exploit the potentials of GEI. The efficiency of the GGE biplot has been compared with other techniques by various cassava researchers (Aina et al., 2009)) for more reliable decisions. The GGE biplot is the most recent and sophisticated among the proposed techniques (Yan et al., 2007). wh This study aimed to evaluate four promising cassava genotypes for fresh root yield and yield components collaboratively with independent commercial cassava processors in the Southeastern region of Nigeria. The results will inform farmers and policymakers about the varieties best suited for the region, thereby supporting demand creation and broader adoption.

## 2 Materials and methods

### 2.1 Study area and locations

**Figure 1:**
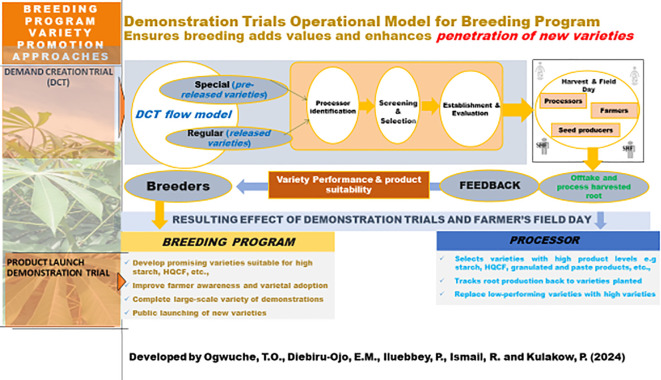
Demonstration trial for operational model for breeding programme.

This study was conducted in three cassava processors’ locations namely Abia state (Von foods), Imo state (Azukamsi and Ebonyi state (Ogbuoji foods) in Southeast Nigeria; Nigeria has three distinct climate zones: a tropical monsoon climate in the South, a tropical savannah climate for most of the central regions, and a Sahelian hot and semi-arid climate in the North. This leads to a gradient of declining precipitation amounts from South to North. Nigeria has an estimated average annual rainfall of 2000 mm, which varies for coastal and inland regions. Southeastern Nigeria is characterized by high temperatures (22-30 degrees centigrade and high humidity of 60-90% throughout the year. Two rainy seasons, March to July and September to November, with a dry season in between. Ultisols and Alfisol are dominant soil types suitable for various crops, including cassava. These areas also serve as processing centers for cassava-based products. The present experiment was conducted at the following locations;

Each location has varying agroecological conditions that reflect the diversity of cassava-growing environments in the region. The research plots at each center were dedicated to cassava cultivation, providing an ideal setting for evaluating improved cassava varieties. The demand creation trial was a demonstration trial created through the Building an Economically Sustainable Integrated Cassava Seed System to promote and introduce improved cassava varieties to processors and farmers. This variety decision support tool is being adopted by the cassava breeding programs at the International Institute of Tropical Agriculture and National Root Crops Research Institute, Umudike, Nigeria, as an annual late-stage variety testing approach. It is making a great impact on the lives of farmers by fostering the adoption of new varieties for different product profiles (Ogwuche et al., 2023). The DCT guarantees food security through sustainable cassava production and processing by creating awareness and promoting new cassava varieties that can rejuvenate the value chain. Eighty-one DCTs were conducted from 2017 to 2024 with different processors, and over 2,070 smallholder farmers were introduced to new varieties through the DCT farmers’ harvest field day. Processors and farmers are currently adopting and demanding improved cassava varieties due to their outstanding performance through the DCT program. Part of the DCT research work was published (Ogwuche et al., 2023). This manuscript is the DCT research activities and data obtained in two consecutive seasons (2021 and 2022) in the Southeastern part of Nigeria. This DCT model is illustrated in **Figure 1** below.

### 2.2 Plant materials

The official names of the improved cassava varieties evaluated in this study are:

- IBA980693
- CR36-5
- TMS13F1160P0004, and
- IBA961632.

These varieties were selected for their high yield potential and resistance to common cassava diseases. TMS13F1160P0004 and IBA980693 were released in 2020 by NextGen cassava projects of the International Institute of Tropical Agriculture and the National Root and Tuber Crops Research Institute, Umudike while IBA961632 and CR36-5 were the older varieties released by the same institutions. These varieties are for three market segments: industry, fresh market, and granulated and paste products. The planting materials for the improved varieties were sourced from the breeder seed field of the National Root Crops Research Institute (NRCRI) in Umudike.

### 2.3 Experimental design

The experiment was laid out in a factorial arrangement, using a randomized complete block design (RCBD) with two replicates at each location. The plot size for each variety 20 m by 16 m with intra and inter-plant spacing of 0.8 m x 1 m, resulting in 400 stands per plot and a net plot of 256 stands. This study was conducted under rain-fed conditions, and no fertilizers were applied. Commercial processors independently managed trials. Recommended weed management routines for cassava were adopted. Harvesting in all locations was performed 12 months after planting (MAP).

### 2.4 Data collection

Measurements of traits: Biotic stress: The severity of Cassava Mosaic Disease (CMD) and Cassava Bacterial Blight Disease (CBB) was scored using the 1 to 5 scale system described by IITA (1 = no symptoms and 5 = severe symptoms). The average CMD and CBB severity were calculated based on ratings taken at 1 and 6 MAP.

Pre-harvest data: At 11 months, data were collected on;

- Plant canopy architecture,
- Plant height,
- Total survival,
- Net survival, and
- Plant vigor.

Harvest data: At 12 months, yield parameters were recorded, including;

- Number of marketable and non-marketable roots,
- Total number of storage roots,
- Root size,
- Weight of marketable and non-marketable root,
- Shoot weight, and
- Bundle weight.

Additional data included;

- Number of rotten roots,
- Root-to-water and
- Root-to-air weight ratios.

Agronomic traits: At 12 MAP, the trials were harvested, and measurements were taken on the number of harvested plants, marketable and non-marketable root number, number with root rot, marketable and non-marketable root weight, number of harvested stem bundles, bundle weight, and shoot weight. Marketable roots are large storage roots, while non-marketable roots are small storage roots. The roots were sampled from each plot across the replications and processed to estimate Dry Matter Content (DMC) following the oven drying method described by Norbert et al.(2019). Fresh Root Yield (FYLD), t/ha, and Dry Matter Content (DMC) per plot were further used to derive the Dry Yield (DYLD) in tonnes per hectare using the expression: DYLD = FYLD/100 x DMC.

### 2.5 Statistical analysis

The data collected across all experimental locations, years, and varieties were subjected to rigorous statistical analyses to evaluate the effects of genotype, environment, and their interactions on agronomic and disease resistance traits in cassava. The study employed a linear mixed model (LMM) framework suitable for multi-environment trials with factorial design. The model structure accommodated the variability across years, locations, and replications while treating genotype (variety) as a fixed effect and other sources of variation as random effects. Specifically, the linear mixed model used was structured as follows:


$$Y_{ijkl}=\mu +G_{i}+L_{j}+Y_{k}+{\left(GL\right)}_{ij}+{\left(GY\right)}_{ik}+{\left(LY\right)}_{jk}+{\left(GLY\right)}_{ijk}+R_{l(jk)}+\in_{ijkl}$$

where:

*Y_ijkl_​ is the observed response for the i^th^ genotype in the j^th^ location, during the k^th^ year, in the l^th^ replicate*;

*μ is the overall mean*;

*G_i_​ is the fixed effect of the i^th^ genotype (cassava variety)*;

*L_j_​ is the random effect of the j^th^ location*;

*Y_k_​ is the random effect of the k^th^ year*;

*(GL)_ij_​, (GY)_ik_​, (LY)_jk_​, and (GLY)_ijk_​ represent random interaction effects*;

*Rl_(jk)_​ is the random effect of the l^th^ replicate nested within location-year*;

*∈_ijkl_​ is the random residual error, assumed to be normally and independently distributed with mean zero and constant variance*.

This modeling approach allowed the partitioning of total phenotypic variance into components attributable to genotype (G), environment (E: location and year), and genotype × environment interactions (G×E), thereby providing insight into the stability and adaptability of the cassava varieties under evaluation. All statistical analyses were performed using the PROC MIXED procedure in R statistical software, which supports complex random and fixed effect modeling. Least squares means (LSMeans) for genotypes were computed and compared using Tukey’s Honest Significant Difference (HSD) test at a significance level of 5%. Where necessary, means were separated using Least Significance Difference (LSD). To further explore genotype performance and stability across environments, Genotype plus Genotype-by-Environment (GGE) biplot analysis was conducted using the R packages agricolae and GGEBiplotGUI, respectively. These multivariate techniques facilitated visualization of interaction patterns, identification of stable genotypes, and specific adaptation to particular locations.

Broad-sense heritability (H²) was calculated for each trait based on the variance components estimated via restricted maximum likelihood (REML) using a linear mixed model. The heritability was computed using the formula:


$$H^{2}=\frac{\sigma_{G}^{2}}{\sigma_{G}^{2}+\frac{\sigma_{GE}^{2}}{e}+\frac{\sigma_{e}^{2}}{re}}$$

Where $\sigma_{G}^{2}$ is the genotypic variance, $\sigma_{GE}^{2}$ is the genotype-by-environment interaction variance, $\sigma_{e}^{2}$ is the residual error variance, e is the number of environments, and r is the number of replications per environment. Variance components were estimated using the ‘lme4’ package in R.

## 3 Results and discussion

### 3.1 Phenotypic evaluation across the environments

**Figure 2:**
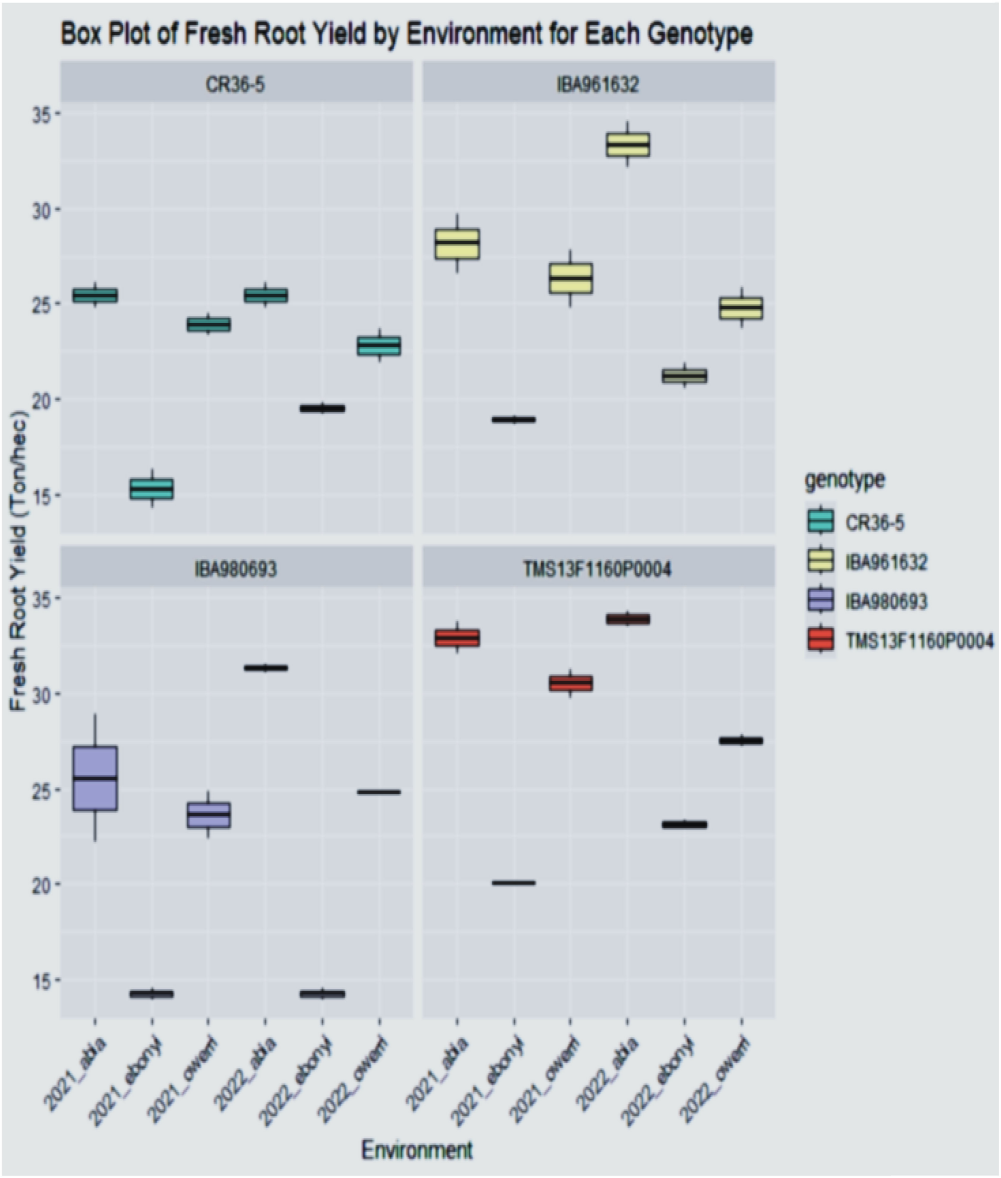
The yield performance distribution of the varieties across the environment.

**Figure 3:**
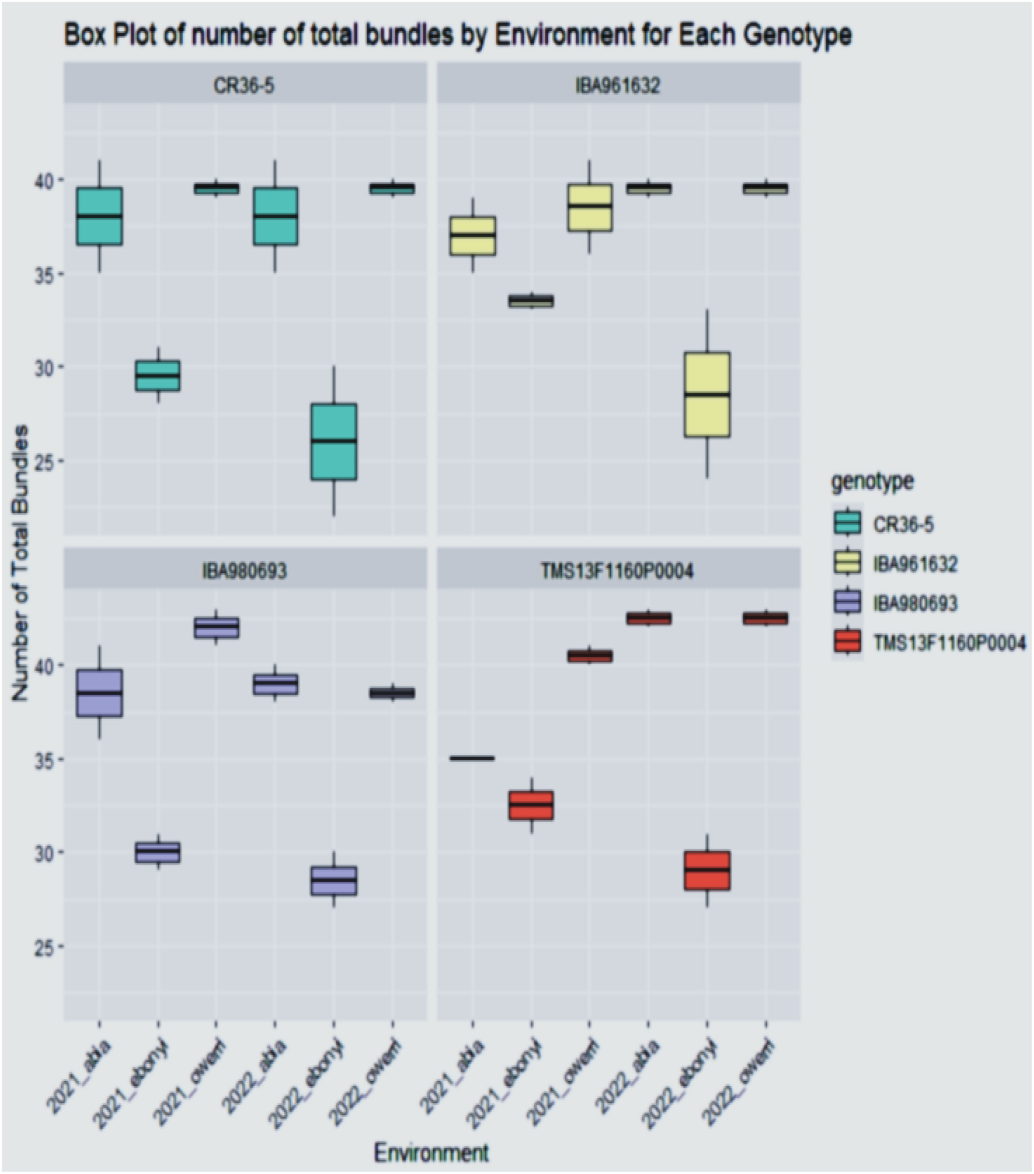
The total bundles estimate the distribution of the varieties across the environment.

**Figure 4:**
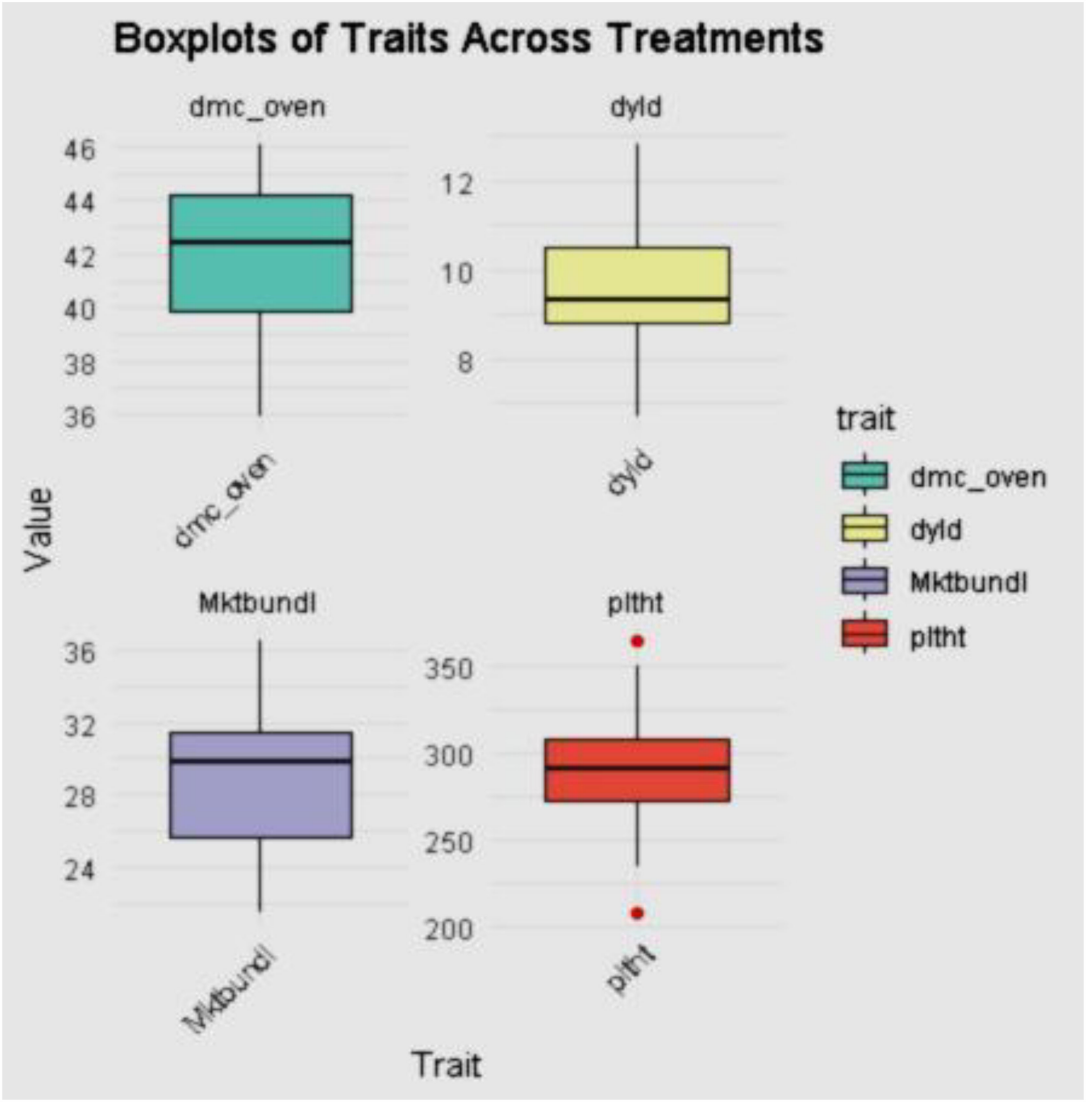
The dry matter, dry yield, marketable bundle, and plant height estimate the distribution of the varieties across the environment.

The phenotypic distribution of the genotypes for fresh root yield performance, total bundle estimate, dry matter content, dry yield, marketable bundle, and plant height is shown as a boxplot in **Figures 2**–**4**. The result showed that the genotype performed significantly different across the environment with genotypes TMS13F1160P0004 and IBA961632 having a higher fresh root yield of 34.5 ton/ha and 34.2 ton/hain Abia location in the year 2022, while the least fresh root yield of 14.5 ton/ha was found in the genotype IBA980693 in Ebonyi location in the year 2021 and 2022.

The total bundle estimate result shown in **Figure 3** revealed that the genotypes have diverse architectural structures. Genotype TMS13F1160P0004 gave the highest total bundles of 45 tiles for Abia and Owerri locations in the year 2022, while the least total bundles measured (26.5 tiles) was found in the genotype CR36-5 in the Ebonyi location in the year 2022. This performance distribution for the yield and the shoot evaluations indicates the inherent attributes of all the genotypes across the trial locations.

The dry matter content, dry yield, marketable bundle estimate, and plant height results across treatments, as shown in **Figure 4**, indicated the diverse differences among the genotypes for the aforementioned traits in this study.

### 3.2 Result of the adjusted yield performance

**Figure 5:**
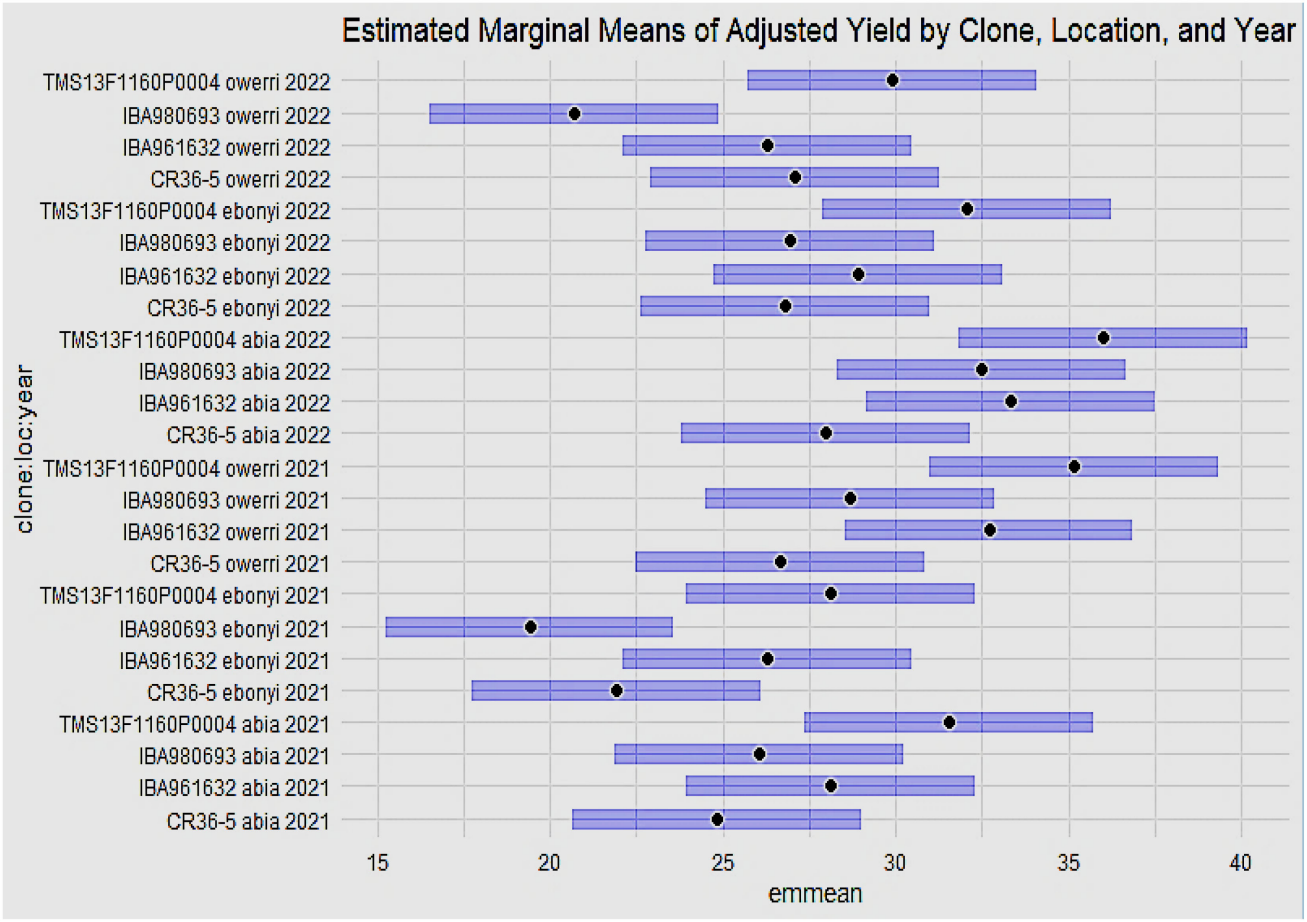
Estimated marginal means of adjusted yield by clone, location and year.

**Figure 5** shows the estimated marginal means of the adjusted yield by clone, location, and year. From the result, genotype TMS13F1160P0004 gave the best overall adjusted yield performance at the Abia location in the years 2021 and 2022, followed by genotype IBA961632, also in the Abia location in the years 2021 and 2022. The least adjusted yield performance was found in genotype IBA980693 at the Ebonyi and Owerri locations in 2021 and 2022, respectively.

**Figure 6:**
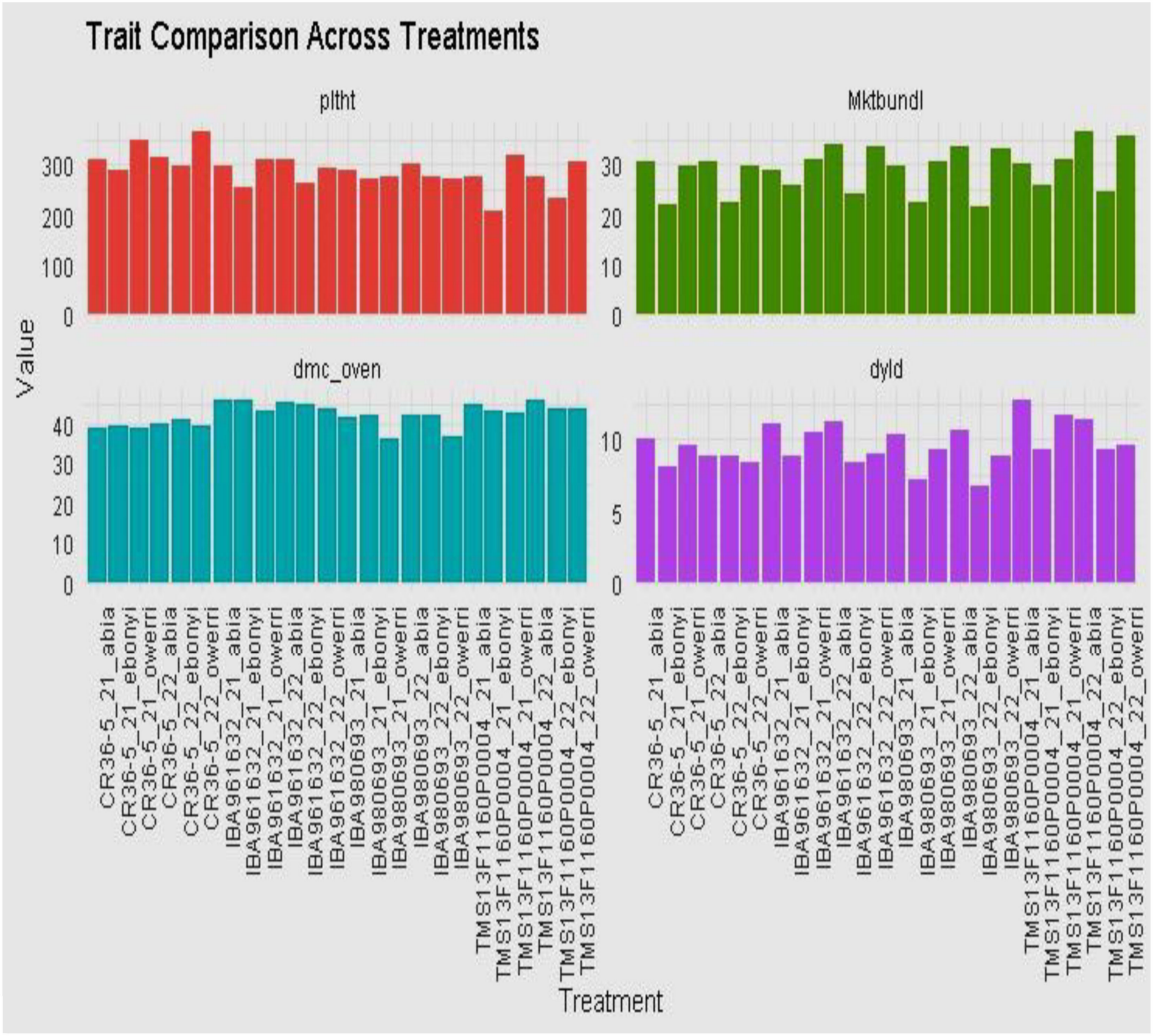
Estimated marginal means of plant height, dry matter content, marketable bundle estimate, and dry yield by clone, location, and year.

Results of plant height, dry matter content, marketable bundles estimate, and dry yield by clone, location, and year are shown in **Figure 6**. Genotype CR36-5 had the highest plant height at Owerri in 2021, and 2022. All the genotypes evaluated across locations and years had average dry matter content of above 25%. Genotypes CR35-5 and IBA961632 had a % dry matter content of 40% in Owerri (2022) and Abia (2021), respectively. The best-performing genotype for dry yield was genotype TMS13F1160P0004 in Owerri in 2021 and Abia in 2022, followed by genotype IBA961632 at the Abia location in 2021.

### 3.3 Mean and variance component of the economic traits

The mean and variance components of the estimated economic traits of the genotypes, showed a significant difference (P<0.05) among the genotypes across the environment (**Tables 1**–**3**). The mean value of the fresh yield ranged from 22.06 to 28.01 ton/haacross the environment; the dry yield ranged from 8.85 to 10.69 ton/ha; dry matter content ranged from 39.62 to 44.79%, bundle estimate ranged from 27.42 to 30.58, the number of roots ranged from 1170 to 1317, plant height ranged from 269.4 to 321.0 cm while the range of the cassava mosaic disease severity is 1.66 to 1.80.

**Table 1:** Test environments, agroecology, and mean annual climatic features.

Year	Location	Temp min.(^0^C)	Coordinates	Temp max. (^0^C)	Rainfall (mm/day)	Relative Humidity (%)
2020	Abia	24.0	lat. 5˚80′N and lon. 7˚53′E	29.0	4.8	87
2020	Imo	23.5	lat. 5˚80′N and lon. 7˚03′E	33.7	4.4	68
2020	Ebonyi	23.8	lat. 5.˚96′N and lon. 7˚77′E	28.8	4.4	82
2021	Abia	25.2	lat. 5˚80′N and lon. 7˚53′E	32.6	5.8	72
2021	Imo	24.0	lat. 5˚80′N and lon. 7˚03′E	34.2	4.6	72
2021	Ebonyi	23.8	lat. 5˚96′N and lon. 7˚77′E	29.8	4.2	68
2022	Abia	22.8	lat. 5˚80′N and lon. 7˚53′E	34.5	4.6	67
2022	Imo	24.2	lat. 5˚80′N and lon. 7˚03′E	34.2	4.2	81
2022	Ebonyi	23.2	lat. 5.˚96′N and lon. 7˚77′E	28.9	4.6	68

**Table 2:** Mean and variance components of the estimated economic traits of the genotypes.

Trait	Mean	δ^2p^	δ^2g^	δ^2e^	H^2^	CV(%)
Fresh yield (t/ha)	28.25	124.46	112.42	10.99	82.99	7.10
Dry yield (t/ha)	9.60	10.06	8.79	2.50	69.98	7.40
Dry matter content (%)	42.14	89.07	84.06	16.40	79.70	3.00
Bundle estimate	29.00	30.50	22.78	4.04	65.95	6.90
No of Root	1231.00	50.30	60.42	17.61	88.97	5.50
Plant height (cm)	289.10	708.39	596.15	4.60	83.65	7.40
CMDS	1.72	86.65	70.07	8.17	73.89	24.10

**Table 3:** Mean estimate of the genotypes across the environment.

Trait	Fresh yield (t/ha)	Dry yield (t/ha)	Dry matter content (%)	Bundle estimate	No of Root	Plant height (cm)	CMDS
CR36-5	22.06	8.99	39.62	27.42	1170	321.0	1.749
IBA961632	25.44	9.87	44.79	29.58	1262	286.7	1.806
IBA980693	22.30	8.85	40.12	28.42	1176	280.4	1.668
TMS13F1160P0004	28.01	10.69	44.02	30.58	1317	269.4	1.715
Grand mean	24.45	9.60	42.14	29.00	1231.4	289.4	1.715
LSD(0.05)	1.247	0.598	1.081	1.693	57.33	18.11	0.3496

The traits exhibited moderate to high broad-sense heritability, with bundle estimate (65.95%) and dry yield (69.98%) reflecting moderate levels, while fresh yield (82.99%), dry matter content (79.70%), number of roots (88.97%), and plant height (83.65%) demonstrated high heritability. These results indicate that, at this stage of evaluation, the traits are largely governed by genetic factors and are therefore considered highly heritable.

### 3.4 Result of the association between the traits

**Figure 7:**
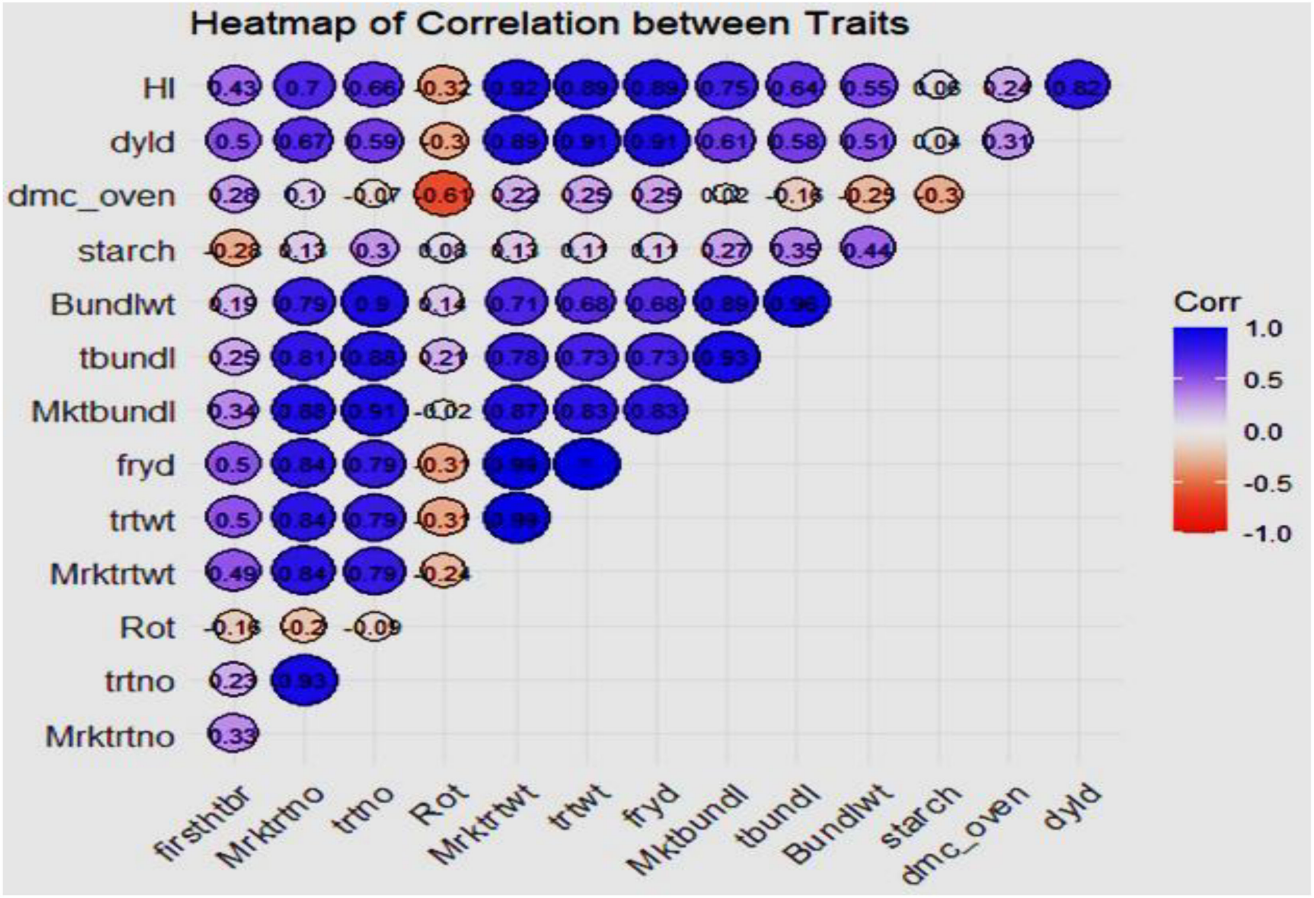
Trait association of the vegetative and yield components of the genotypes across the environment.

The heatmap in **Figure 7** shows the correlation between various traits, with each cell representing the correlation coefficient (ranging from -1 to 1) between a pair of traits. Each cell’s color and size indicate the correlation’s strength and direction, where dark blue cells represent strong positive correlations (close to 1). Light blue or white cells represent weak or no correlations (close to 0). Red cells represent negative correlations (close to 1).

### 3.5 Result of the genotype by environment biplot for four cassava genotypes in processors sites

**Figure 8:**
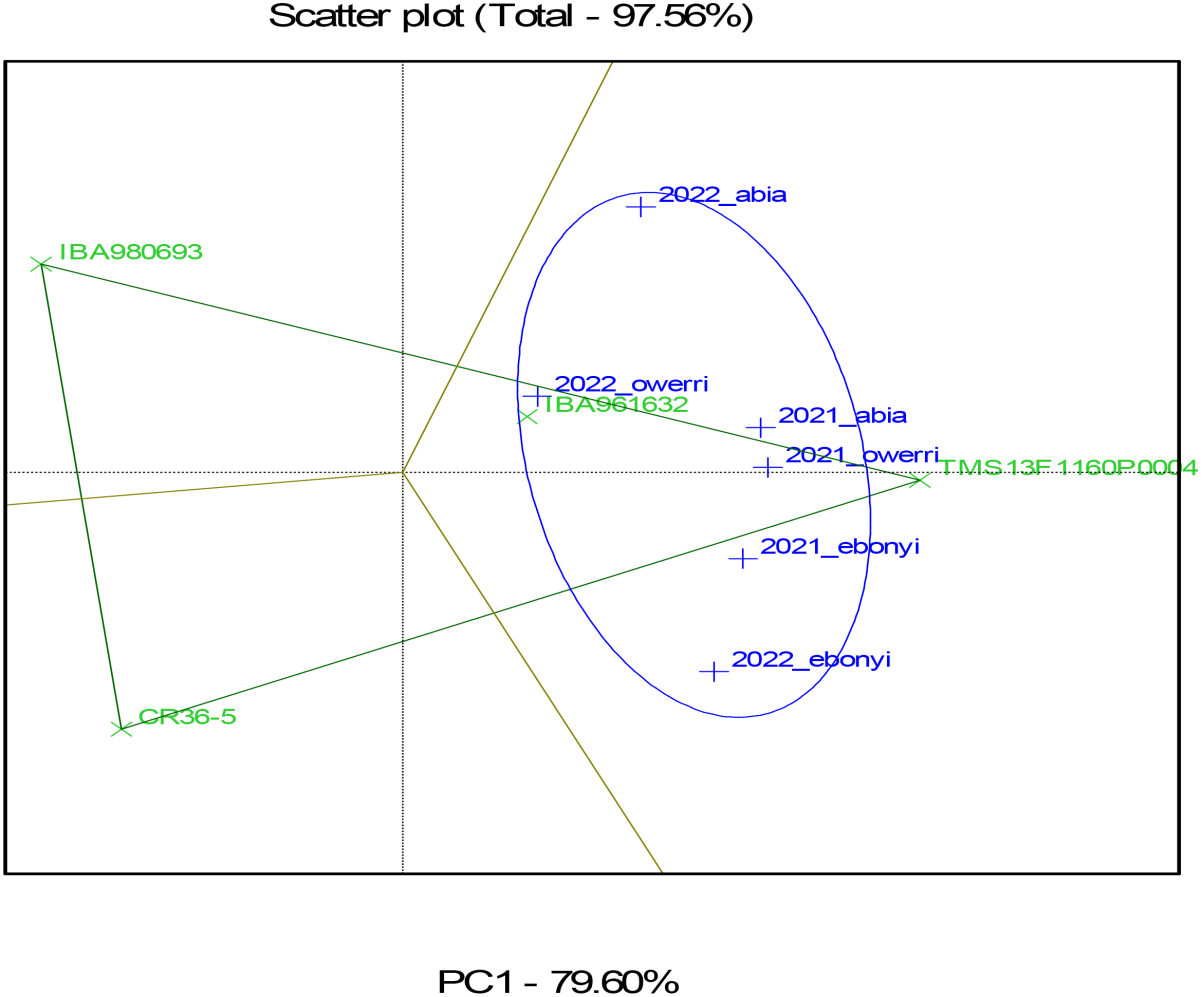
GGEBiplot of fresh root yield of the genotypes across location and year.

**Figure 9:**
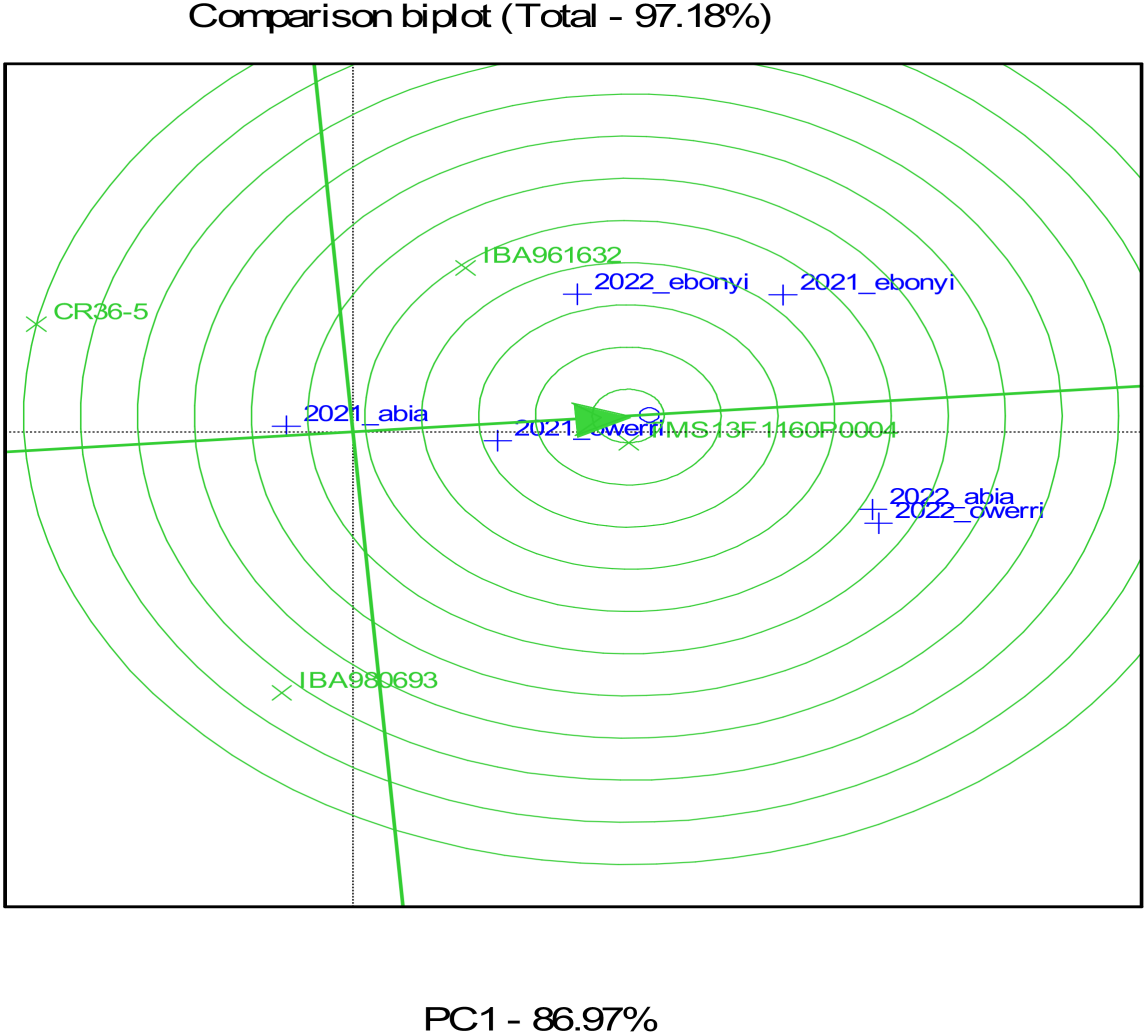
GGEBiplot of dry matter content of the genotypes across location and year.

**Figure 10:**
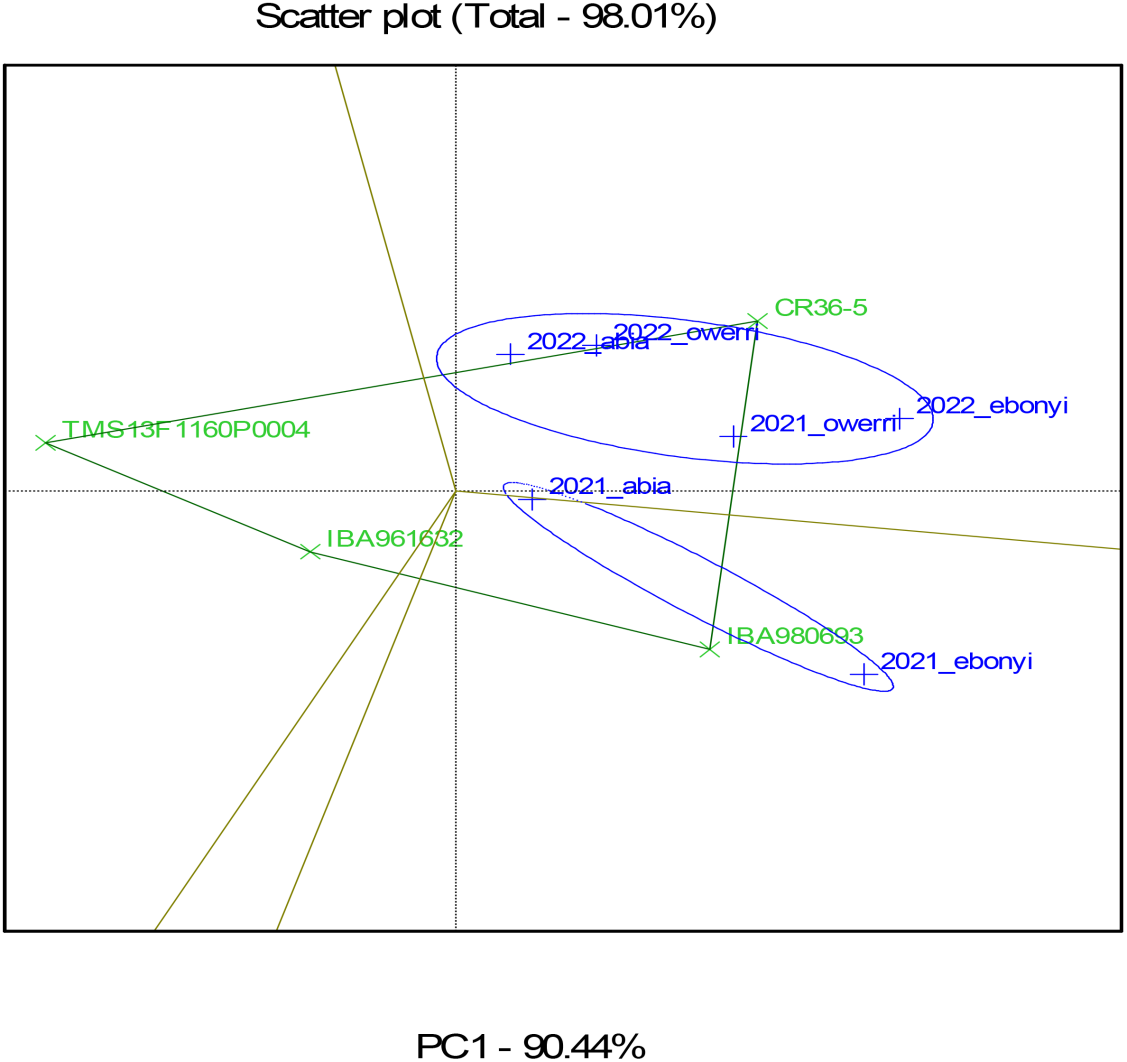
GGEBiplotof the bundle estimate of the genotypes across location and year.

**Figures 8**–**10** represent Genotype by Environment (GxE) Biplot analyses, typically used to evaluate the performance and stability of genotypes across multiple environments for the fresh root yield, dry matter content, and bundle estimate. The biplot’s axes represent principal components (PC1 and PC2), which capture the most significant variation in these traits due to genotype and environment interaction. Each vector (line) from the origin (center) to a genotype or environment point represents the effect of that genotype or environment on fresh root yield. The green color highlights the genotypes, showing their performance and interaction patterns across multiple environments. The blue color identifies the test environments and marks the confidence limits within which the majority of environmental points are contained. Genotypes or environments positioned farther from the origin indicate more significant contributions to variation. The ellipse encompasses similar environments in terms of their interaction with genotypes. Environments within this circle have similar response patterns, suggesting they exert similar environmental pressures or conditions. Genotypes closer to certain environments within the circle, as seen in TMS13F1160P0004 near 2021_owerri and 2022_abia, are likely to perform better in these environments, showing an adaptation or preference.

From the result, Genotypes TMS13F1160P0004 and IBA961632, situated closer to the origin, may be more stable across environments, meaning they perform consistently regardless of environmental differences. Conversely, genotypes located farther from the origin, such as CR36-5, may perform exceptionally well in specific environments but might be more sensitive to environmental changes. Environments such as 2021_owerri and 2022_abia in fresh root yield that form a wide angle with each other or have long vectors are more discriminating, meaning they better differentiate genotypes. Environments closer to the average environment axis (closer to the origin) may be more representative of the overall conditions across locations.

The result of the GGE biplot shows that TMS13F1160P0004 is the most stable genotype regarding dry matter content, fresh root yield, and estimated bundle across the tested environments. Genotypes like CR36-5 and IBA980693, being farther from the center, are more influenced by environmental conditions, indicating they might perform well in specific environments but lack consistency across all tested conditions. This information can help breeders select genotypes with stable performance for dry matter content across diverse environments.

## 4 Discussion

The phenotypic distribution of fresh root yield (FRY) and total bundle estimate (TBE) across environments reveals substantial variability among the genotypes. Genotypes TMS13F1160P0004 and IBA961632 exhibited the highest fresh root yield, achieving 34.5 t/ha and 34.2 t/ha, respectively, in Abia in 2022. Conversely, IBA980693 recorded the lowest performance of 14.5 t/ha in Ebonyi during both 2021 and 2022. Such variation highlights significant genotype-by-environment interactions, as observed in other studies where genotypic adaptability and environmental influence shaped yield outcomes (Okechukwu et al., 2019; Asante et al., 2021). The diversity in Total Bundle Estimate (TBE) among the genotypes showed that genotype TMS13F1160P0004 recorded the highest total bundles (45 tiles) in Abia and Owerri in 2022, suggesting a robust architectural structure. In contrast, CR36-5 showed the lowest TBE (26.5 tiles) in Ebonyi in 2022. These variations reflect the influence of environmental conditions on the development of cassava canopy architecture, as previously noted by Anikwe and Ikenganyia (2018). The fresh root yield (**Figure 5**) further substantiates the superior performance of TMS13F1160P0004 and IBA961632 in Abia during 2021 and 2022, while IBA980693 remained the poorest performer in Ebonyi and Owerri during the same period. Mean and variance analysis (**Tables 2**–**4**) revealed statistically significant differences (P < 0.05) among genotypes across environments. Fresh root yield ranged from 22.06 to 28.01 t/ha, dry yield from 8.85 to 10.69 t/ha, and dry matter content from 39.62% to 44.79%. These results align with reports by Egesi et al. (2017), emphasizing genotype-specific performance and environmental adaptation in cassava.

**Table 4:** Variance component of the individual and interactive effect across genotypes and environment .

Source of Variation	DF	Fresh yield (t/ha)	Dry yield (t/ha)	Dry matter content (%)	Bundle estimate	Number of Root	Plant height (cm)	CMDS
Genotype (G)	3	95.95***	8.79***	84.79***	22.78**	60420**	5961.5***	0.0699
Location (L)	2	515.03***	24.40***	31.95***	346.67***	286469***	10442.6***	1.0149**
Year (Y)	1	24.30**	4.80**	4.26	36.75**	49665**	300.0	0.1875
G x L	6	8.22**	1.26*	7.82**	2.30	5457	1887.4**	0.1583
G x Y	3	2.42	0.52	1.35	4.53	3634	15.6	0.0212
L x Y	2	19.87**	1.73*	0.18	24.94	12362	303.5	0.1875
G x L x Y	6	6.15*	0.55	0.45	3.88**	9669	152.50.	0.0212

Significant codes: ‘***’ 0.001 ‘**’ 0.01, ‘*’ 0.05; DF, degree of freedom; CMDS, cassava mosaic disease severity.

The study revealed that the evaluated traits exhibited moderate to high broad-sense heritability, suggesting a strong genetic influence on their expression. Specifically, bundle estimate (65.95%) and dry yield (69.98%) demonstrated moderate heritability, while traits such as fresh yield (82.99%), dry matter content (79.70%), number of roots (88.97%), and plant height (83.65%) showed high heritability estimates. High heritability indicates that a significant proportion of the observed phenotypic variance can be attributed to genotypic variance, implying that these traits are primarily governed by additive genetic effects and are less influenced by environmental factors (Falconer and Mackay, 1996; Hallauer et al., 2010). These findings are consistent with previous reports in cassava (Esuma et al., 2016; Oliveira et al., 2012) and yam (Asfaw, 2011; Mignouna et al., 2002), where yield-related and morphological traits were found to possess heritability estimates within similar ranges.

The heat map (**Figure 7**) illustrates trait correlations, revealing strong positive relationships between fresh root yield (FRY) and total root weight (r = 0.99), as well as between marketable bundle weight and total stem bundles (r = 0.96). Conversely, dry matter content exhibited a moderate negative correlation with dry root yield (r = -0.6), indicating an inverse relationship. These findings align with Akinwale et al. (2018), who observed trait interdependencies impacting cassava breeding goals. Harvest index (HI) and dry root yield (dyld) showed a relatively high positive correlation (r = 0.82), which could imply that as the harvest index increases, the dry yield tends to increase as well. The figure also shows that dry root yield (dyld) and total stem bundle (tbundl) had moderate positive correlations (r = 0.73). Dry matter content (dmc_oven) and dry root yield (dyld) had a moderate and negative correlation (r = -0.6), which may indicate an inverse relationship. This biologically may be attributed to the fact that plants may allocate resources to either root growth or dry matter accumulation, leading to a trade-off between the two traits or environmental conditions, such as water availability or nutrient supply, can impact root growth and dry matter accumulation differently. The timing and method of harvesting can also impact dry matter content and root yields, in addition to processing and storage techniques. This may be the reason for the inconsistent inverse correlation between dry matter content and dry yields observed in this study. **Figures 8**–**10** depict GxE biplots for fresh root yield, dry matter content, and total bundle estimate. Principal component axes (PC1 and PC2) capture most of the variation due to genotype and environmental interactions. The IITA cassava breeding program continually evaluates many clones in several target locations over years aiming to identify clones with high yield productivity and stability and to assess adaptability across a wide range of diverse environmental conditions. This evaluation necessitates the establishment of multi-environment trials annually to determine the clones’ yield performance across various agro-ecological zones in Nigeria. The release of new varieties arises when the clones possess specific characteristics that prove their desirable performance for a given geographical region, emphasizing the importance of MET in plant breeding programs (Oliveira et al., 2020). TMS13F1160P0004 and IBA961632, positioned near the origin, were identified as the most stable genotypes across environments. These results align with reports by Ogwuche et al. (2023), identifying three varieties, namely IBA961632, TMEB419, and CR36/5, as most promising for dry yields and related traits by cassava processors in Nigeria, and genotype IBA961632 was the most stable. Environments such as 2021_Owerri and 2022_Abia were more discriminating, effectively distinguishing genotype performance. Conversely, CR36-5 and IBA980693, positioned farther from the origin, showed high sensitivity to environmental variations. Similar findings by Yan and Tinker (2006) highlighted GxE biplots as a critical tool for identifying stable genotypes and discriminating environments in multi-environment trials. This justifies the need for further analysis to probe into GEI and integrate yield performance with stability across locations for a reliable decision on the superiority or desirability of the genotypes.

## 5 Conclusion

This work identified TMS13F1160P0004 and IBA961632as the most stable and high-yielding genotypes across traits. These genotypes demonstrated wide adaptability, making them prime candidates for deployment in diverse agroecological zones.While genotype CR36-5 and IBA980693 were observed to be unstable and low-yielding, they demonstrated specific adaptability, they may require targeted environmental conditions for optimal performance. The GxE analysis provides valuable insights for breeders aiming to balance high-yield potential with broad adaptability in cassava.

Based on the findings, genotypes TMS13F1160P0004 and IBA961632 should be prioritized for large-scale cultivation due to their superior fresh root yield, dry matter content, and consistent performance across multiple environments. These genotypes also demonstrated stability in key traits such as estimated bundle size and plant architecture, making them suitable for diverse agroecological conditions in Sub-Saharan Africa, especially countries that shared similar climatic features with the study sites Genotypes such as CR36-5 and IBA980693, which performed well in specific environments but lacked consistency, can be strategically deployed in environments like Ebonyi and Owerri, where their unique traits align with environmental conditions. Targeted cultivation in favorable locations will maximize their productivity and minimize risks from environmental sensitivity.

The study highlights the importance of Genotype-by-Environment (GxE) interaction analyses for identifying adaptable and stable genotypes. Breeding programs should adopt GxE biplot or other approaches to refine selection indices and ensure newly developed genotypes are resilient to varying environmental conditions. This will enhance genetic gain and improve breeding efficiency. Strong positive correlations between traits such as fresh root yield, dry root yield, and total bundle weight suggest that breeders should use these traits as proxies for selecting high-performing genotypes. Emphasis should also be placed on traits like dry matter content and harvest index to meet market demands for processing and consumer preference, ensuring product quality and economic viability.

## Data Availability

The datasets presented in this study can be found in online repositories. The names of the repository/repositories and accession number(s) can be found below: The datasets generated and/or analyzed during the present study can be found in the Cassava base repository https://www.cassavabase.org.

## References

[B1] Abbo S. Lev-Yadun S. Gopher A. (2010). Yield stability: An agronomic perspective on the origin of Near Eastern agriculture. Vegetation History Archaeobotany 19, 143–150. doi: 10.1007/s00334-009-0233-7

[B2] Acheampong P. P. Addison M. Wongnaa C. A. (2022). Assessment of the impact of adoption of improved cassava varieties on yields in Ghana: An endogenous switching approach. Cogent Economics Finance 10, 2008587. doi: 10.1080/23322039.2021.2008587

[B3] Aina O. O. Dixon A. G. O. Paul I. Akinrinde E. A. (2009). G×E interaction effects on yield and yield components of cassava (landraces and improved genotypes) in the savannah regions of Nigeria. Afr. J. Biotechnol. 8, 4933–4945. Available online at: https://www.ajol.info/index.php/ajb/article/view/61090

[B4] Akinwale M. G. Aladesanwa R. D. Akinyele B. O. Dixon A. G. O. (2018). Genetic variability and heritability estimates in cassava genotypes under different agro-ecologies in Nigeria. J. Crop Improvement 32 (3), 366–381. doi: 10.1080/15427528.2018.1449003

[B5] Anikwe M. A. Ikenganyia E. E. (2018). Ecophysiology and production principles of cassava (Manihot species) in Southeastern Nigeria. In Akinwale A. A. (Ed.), Cassava. IntechOpen. 105–122. doi: 10.5772/intechopen.69424

[B6] Annicchiarico P. Mariani G. (1996). Prediction of adaptability and yield stability of durum wheat genotypes from yield response in normal and artificially drought-stressed conditions. Field Crops Res. 46, 71–80. doi: 10.1016/0378-4290(95)00087-9

[B7] Asante I. K. Offei S. K. Danquah E. Y. Blay E. T. Egesi C. (2021). Genetic analysis of yield and quality traits in cassava. Agron. J. 113 (2), 1234–1245. doi: 10.1002/agj2.20567

[B8] Asfaw A. (2011). Genetic improvement of cassava for resistance to viral diseases in Ethiopia. Afr. Crop Sci. J. 19 (4), 345–356.

[B9] Ceccarelli S. (1996). “Positive interpretation of genotype by environment interaction about sustainability and biodiversity,” in Plant adaptation and crop improvement. Eds. Cooper M. Hammer G. L. (CABI, Wallingford, UK), 467–486.

[B10] Diagne A. (2009). Technological change in smallholder agriculture: Bridging the adoption gap by understanding its source (University of California: eScholarship).

[B11] Egesi C. N. Ogunolu F. D. Okechukwu R. U. Okogbenin E. (2017). Breeding cassava for multiple pest and disease resistance in Africa. Crop Sci. 57 (5), 2377–2389. doi: 10.2135/cropsci2016.12.0989

[B12] Esuma W. Kawuki R. Herselman L. Labuschagne M. (2016). Genetic diversity of provitamin A cassava in Uganda. J. Agric. Sci. 154 (4), 679–691. doi: 10.1017/S0021859615000682

[B13] Falconer D. S. Mackay T. F. C. (1996). Introduction to quantitative genetics (4th ed.) (Longman).

[B14] Food and Agriculture Organization of the United Nations (2023). Agricultural production statistics (Rome, Italy: Food and Agriculture Organization of the United Nations).

[B15] Hahn S. K. Terry E. R. Leuschner K. Akobundu I. O. Okali C. Lal R. (1979). Cassava improvement in africa. Field Crops Res. 2, 193–226. doi: 10.1016/0378-4290(79)90024-8

[B16] Hallauer A. R. Carena M. J. Miranda Filho J. B. (2010). Quantitative genetics in maize breeding (3rd ed.) (Springer). doi: 10.1007/978-1-4419-0766-0

[B17] Hershey C. H. (1988). “Cassava breeding- CIAT headquarters,” in Cassava breeding and agronomy research in Asia (Cali, Colombia: Centro Internacional de Agricultura Tropical (CIAT)), 99–116.

[B18] Kalkuhl M. Von Braun J. Torero M. (2016). “Volatile and extreme food prices, food security, and policy: an overview,” in Food price volatility and its implications for food security and policy (Cham, Switzerland: Springer International Publishing), 3–31.

[B19] Kvitschal M. V. Vidigal Filho P. S. Scapim C. A. Gonçalves-Vidigal M. C. Pequeno M. G. Sagrilo E.. (2006). Evaluation of phenotypic stability of cassava clones by AMMI analysis in northwestern Paraná state. Crop Breed. Appl. Biotechnol. 6, 236–241. doi: 10.12702/1984-7033.v06n03a08

[B20] Lin Z. J. D. Taylor N. J. Bart R. (2019). Engineering disease-resistant cassava. Cold Spring Harbor Perspect. Biol. 11, a034595. doi: 10.1101/cshperspect.a034595 PMC6824239 31182545

[B21] McCallum E. J. Anjanappa R. B. Gruissem W. (2017). Tackling agriculturally relevant diseases in the staple crop cassava (Manihot esculenta). Curr. Opin. Plant Biol. 38, 50–58. doi: 10.1016/j.pbi.2017.04.008 28477536

[B22] Mignouna H. D. Dixon A. G. O. Thottappilly G. (2002). Genetic diversity of cassava (Manihot esculenta Crantz) landraces and cultivars from southern Nigeria. J. Genet. Breed. 56 (2), 171–180.

[B23] Mühleisen J. Piepho H.-P. Maurer H. P. Longin C. F. H. Reif J. C. (2014). Yield stability of hybrids versus lines in wheat, barley, and triticale. Theor. Appl. Genet. 127, 309–316. doi: 10.1007/s00122-013-2219-1 24162154

[B24] Mwebaze P. Macfadyen S. De Barro P. Bua A. Kalyebi A. Bayiyana I.. (2024). Adoption determinants of improved cassava varieties and intercropping among East and Central African smallholder farmers. J. Agric. Appl. Economics Assoc. 3, 1–15. doi: 10.1002/jaa2.v3.2

[B25] Nassar N. M. Ortiz R. (2006). Cassava improvement: Challenges and impacts. J. Agric. Sci. 145, 163–171. doi: 10.1017/S0021859606006575

[B26] Ogwuche T. O. Diebiru-Ojo M. E. Najimu A. Ossai C. O. Ekanem U. Adegbite B.. (2023). Performance and stability of improved cassava (*Manihot esculenta* Crantz) clones in demand creation trials in Nigeria. Crops 3, 209–219. doi: 10.3390/crops3030020

[B27] Okechukwu R. U. Egesi C. N. Kulakow P. Offei S. K. (2019). Genetic improvement of cassava for enhanced yield and related traits in Nigeria. Plant Breed. 138 (3), 321–330. doi: 10.1111/pbr.12689

[B28] Oliveira E. J. Ferreira C. F. Santos V. S. Jesus O. N. (2012). Genetic parameters and prediction of genotypic values in cassava using REML/BLUP. Genet. Mol. Res. 11 (2), 1319–1329. doi: 10.4238/2012.May.15.4 25177949

[B29] Oliveira I. C. M. Guilhen J. H. S. Ribeiro P. C. D. O. Gezan S. A. Schaffert R. E. Simeone M. L. F.. (2020). Genotype-by-environment interaction and yield stability analysis of biomass sorghum hybrids using factor analytic models and environmental covariates. Field Crop Res. 257, 107929. doi: 10.1016/j.fcr.2020.107929

[B30] Udensi O. Ikpeme E. V. Edu E. A.. (2011). Adoption of selected improved cassava varieties among smallholder farmers in South-Eastern Nigeria. J. Food Agric. Environ. 9, 329–335.

[B31] Yan W. (2001). GGEbiplot—A Windows application for graphical analysis of multienvironment trial data and other types of two-way data. Agron. J. 93, 1111–1118. doi: 10.2134/agronj2001.9351111x

[B32] Yan W. Kang M. S. Ma B. Woods S. Cornelius P. L. (2007). GGE biplot vs. AMMI analysis of genotype-by-environment data. Review and interpretation. Crop Sci. 47, 641–653. doi: 10.2135/cropsci2006.06.0374

[B33] Yan W. Tinker N. A. (2006). Biplot analysis of multi-environment trial data: Principles and applications. Can. J. Plant Sci. 86, 623–645. doi: 10.4141/P05-169

