# Supplementary material for: Evaluation of improved cassava genotypes for fresh root yield and yield components in demand creation trial
Source: Front Plant Sci. 2025 Jun 6;16:1564393. doi: 10.3389/fpls.2025.1564393 (PMC12179145; doi:10.3389/fpls.2025.1564393)
Supplement: Supplementary file 1 [file DataSheet1.pdf]

Demand Creation Trial: Information Tool Variety Development and Adoption Scheme for Breeder, Processor, and Farmer

Introduction

As Nigeria increases participation in the cassava global market due to growth in the processing of the crop into other secondary industrial products such as starch, flour, ethanol, animal feed, glucose syrup, etc., the need to identify varieties well suited for the diverse product purpose becomes eminent. Although a huge percentage of its production still lies internally in the hands of small-scale farmers. In Nigeria, so far 56 improved varieties are officially released out of which less than 10 are popular among farmers and other users. These cassava varieties are used across the different products which does not maximize the quality trait they possess. Cassava root farmers have benefitted little in the sale of roots to industries and processors because they don't have the required varieties. Starch and flour processors will look out for varieties with high dry matter content and starch content in addition to fresh root yield. Garri-making processors will look out more for traits such as yield and sometimes the carotenoid contents for the biofortified materials so also for livestock feed processors. This brings about the importance of product profiling of cassava. Demand Creation Trials is a decision-making tool developed by the IITA Cassava breeding program in collaboration with Context Global Development under the BASICS1 project. Processors identified by Context Global Development are engaged to conduct the Demand Creation Trial (DCT) and make product decisions like the selecting preferred variety for their production and making production plans. The DCT is the large trial of 3-10 improved and released varieties established in the processor's fields. The fields are harvested, and varieties are processed separately into the products of the industry, after which data on the collected, evaluated, and ranked.

Snapshot: DCT PROCESS MODEL

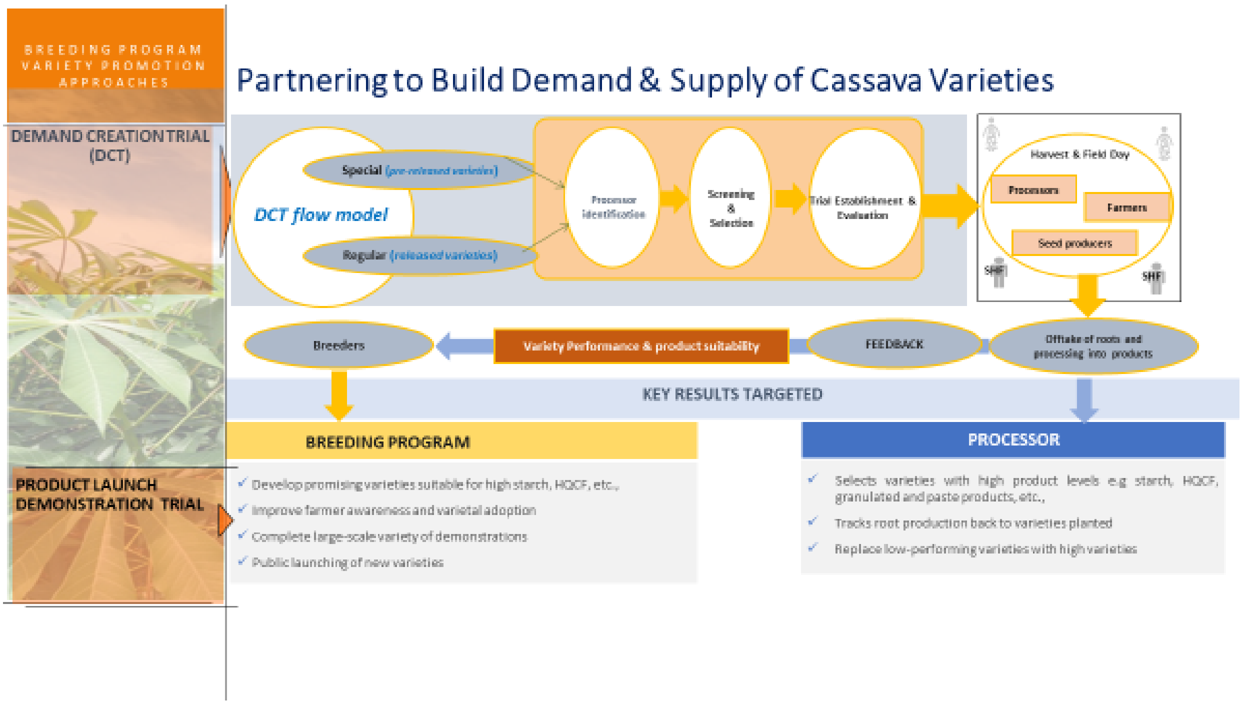

Steps

Step	Activity	Description
1	Identify New Processor	Send a mail or make a phone call to potential processor who will clearly indicate his/her land size, field location, product lines and varietal preference if there is any
2	Carry Out Field and Factory Inspection	Research supervisor would be deployed to inspect the state of factory, production capacity and fields for suitability for DCT program
3	Draft and share MoU	If number two condition is met, prepared MoU will be share with processor to official seal the agreement for both party
4	Trial Establishment and Traning on GAPS	Training on GAPs tailored specifically to root production would be provided to processor's agronomist or farm manager to improve processor' yield in addition to planting coordination
5	Field monitoring and Periodic Trial Evaluation	Virtual field monitoring on WhatsApp platform to ensure quality control. In addition will be periodic field evaluation at one and six months after planting for growth data collection
6	Harvest Field Day Feedback Adoption	Collaborate with processor to invite farmers and other processors for final trial harvest to demonstrate performance of new varieties. Feedback from participants on variety performance and suitability will be obtain

Procedural Concept of DCT

Demand Creation Trial (DCT) has been proven to be a useful decision tool for the breeder, seed producer, large-scale processor, and farmer. It is serving as an important source of evidenced information for breeders. Hence, need for long-term adoption of DCT by agro-enterprises'. Under the scheme, the processor/seed producer/farmer shall grow improved cassava varieties as recommended by IITA by the product of interest of the processor/farmer. Planting materials shall be supplied by the cassava breeding unit of IITA.

1. **Annual DCT arrangement**
    a. IITA/NRCRI decides on the number of processors to engage annually depending on available resources.
    b. Identification of suitable processors or engagement of existing processors across the various Nigeria agro-ecological zones. These processors are grouped into; large, medium, and small-scale processors
    c. Processors are classified as new or existing
    d. DCT is termed regular or special depending on the type and purpose e.g nextgen, harvest plus and regular for dry yield, biofortification, and both respectively
    e. IITA/NRCRI breeders are responsible for the annual nomination of varieties to be tested within the market-driven and candidate lines. However, existing processors can request for inclusion of preferred variety into the new list
    f. Agreement DCT documents are sent to qualify processors indicating the date, design, land area, number of varieties, and others

2. **Trial layout**
    a. Land preparation either ridge or flat (preferably ridge)
    b. Replicated three times
    c. Spacing 0.8m by 1m
    d. Serpentine arrangement
    e. Gross plot 300sqm and net plot 234sqm (15m x 15m)
    f. Land area depending on the number of varieties but not more than 2.5 acreage
    g. Randomised Incomplete block (large plot utilizing trial)

3. **Obligations of Processor/seed producer**
    a. Provision of land measuring a minimum of x hectare at a location and conforming to standards agreeable to IITA, for the planting and arrangement of all operations relating to land preparation.
    b. Provision of physical labour for field establishment under the supervision of IITA and NRCRI.
    c. Responsible for total field maintenance and other input applications and shall strictly follow IITA standard field management operation
    d. Responsible for the cost and operations of harvesting.
    e. Safeguard the trial
    f. Allow IITA and NRCRI teams access to the field for close monitoring, inspection, evaluation, and supervision visit at as when due without restriction.
    g. Provide information for farmer's evaluation form which will consist of questions regarding field establishment, agronomy, plant architecture, diseases etc. while processors evaluation forms will be focused on root characteristics, dry matter, processing properties, product quality etc. during and after root harvest
    h. Offtake the harvested roots, processed into preferred products and share the result of each variety performance with IITA.
    i. Provide IITA, feedback on suitability of the varieties tested in line with their product at the end of the trial
    j. Make seed order to Umudike Seeds and IITA GoSeed with respect to selected variety and required quantity

4. **Obligations of IITA and NRCRI**
    a. Responsible for the cost of the stem and the transportation of the stem to processor destination
    b. Provide require cassava stakes and varieties to be tested depending on the type of DCT (regular, nextgen and harvestplus)
    c. Conduct periodical visits to trial site and shoulder all cost attributed to this activity
    d. Collect data major pest and disease scoring, growth and yield performance ratings
    e. Give at least one week notice to processor prior to harvest
    f. Administer evaluation forms to farmers or processors during root harvest which shall be duly filled
    g. Share data and results from findings with partners on request

5. **Conditions for Harvesting**
    a. Harvesting shall only be undertaken under the strict supervision of IITA and NRCRI.
    b. At harvest, the field shall be measured by IITA and NRCRI to get the specified requirement.
    c. 100% of the roots shall be taken by the processor/seed producer/farmer.
    d. Harvested stems shall be used for new trial establishment.

Key Outcome

- The DCT helped Shao farms make decisions on what to produce in the SAH lab to maximize cost and production
- Alternative to known and common varieties varieties are identified for example IBA 961632 a close substitute for TME419 while Baba 70 was identified as a variety that replaced Fine face.
- Game changer has been identified to have exhibited high level of dry matter consistently and its stable.
- Dry matter content, a key trait for processors is known in specific locations/processors fields
- Appropriate time for harvest to obtain good values for dry matter and starch in known
- Varieties that maintain high and stable dry matter are noted
- Improved varieties such as Ayaya, Baba 70 and farmer's pride were identified as varieties with improve shelf life by farmers and processors. Obasanjo 2 was selected for high yielding and late maturing variety.
- Dixon was identified as a drought tolerant variety that can mitigate the effect of climate change especially in northern Nigeria.
- DCT information has helped breeders to understand farmers and processors preferences. This lead to demand-led breeding service that can foster adoption of new varieties. It is serving as a strong evidence base decision support tool for stakeholders in the cassava value chain and subsistence farmers.

Summary, Conclusions and Publication

- DCT was used as a tool to deliver improved high-quality and nutritious cassava varieties to over 65 processors and 1,070 farmers. Stimulate adoption and increase genetic gain in farmers' fields to improve the livelihood of small holders' farmers. Hence the need for the breeding program at IITA and NARS to adopt this system as a pathway for fast delivery of improve and nutritious varieties of cassava to farmers' fields in Africa sustainably.
- Farmers and Out growers should participate more in the trials, this will help them understand the market varieties they grow, hence need to conduct more DCTs by the cassava breeding program in order to accommodate more farmers.
- Every processor should have a DCT in his/her field and make it part of the organizations operations. This system will help processors to develop a sustain out grower farmers model who will accept stems of improved varieties.
- Need for breeding program to adopt the concept of DCT to delivery to farmers high-quality, climate-resilient, market-preferred, and nutritious varieties of cassava while ensuring equitable access for women and other disadvantaged groups
- https://www.mdpi.com/2673-7655/3/3/20
- https://www.mdpi.com/2673-7655/3/3/20/pdf
