# Supplementary material for: Evaluation of improved cassava genotypes for fresh root yield and yield components in demand creation trial
Source: Front Plant Sci. 2025 Jun 6;16:1564393. doi: 10.3389/fpls.2025.1564393 (PMC12179145; doi:10.3389/fpls.2025.1564393)
Supplement: Supplementary file 2 [file Presentation1.pptx]

## Slide 1
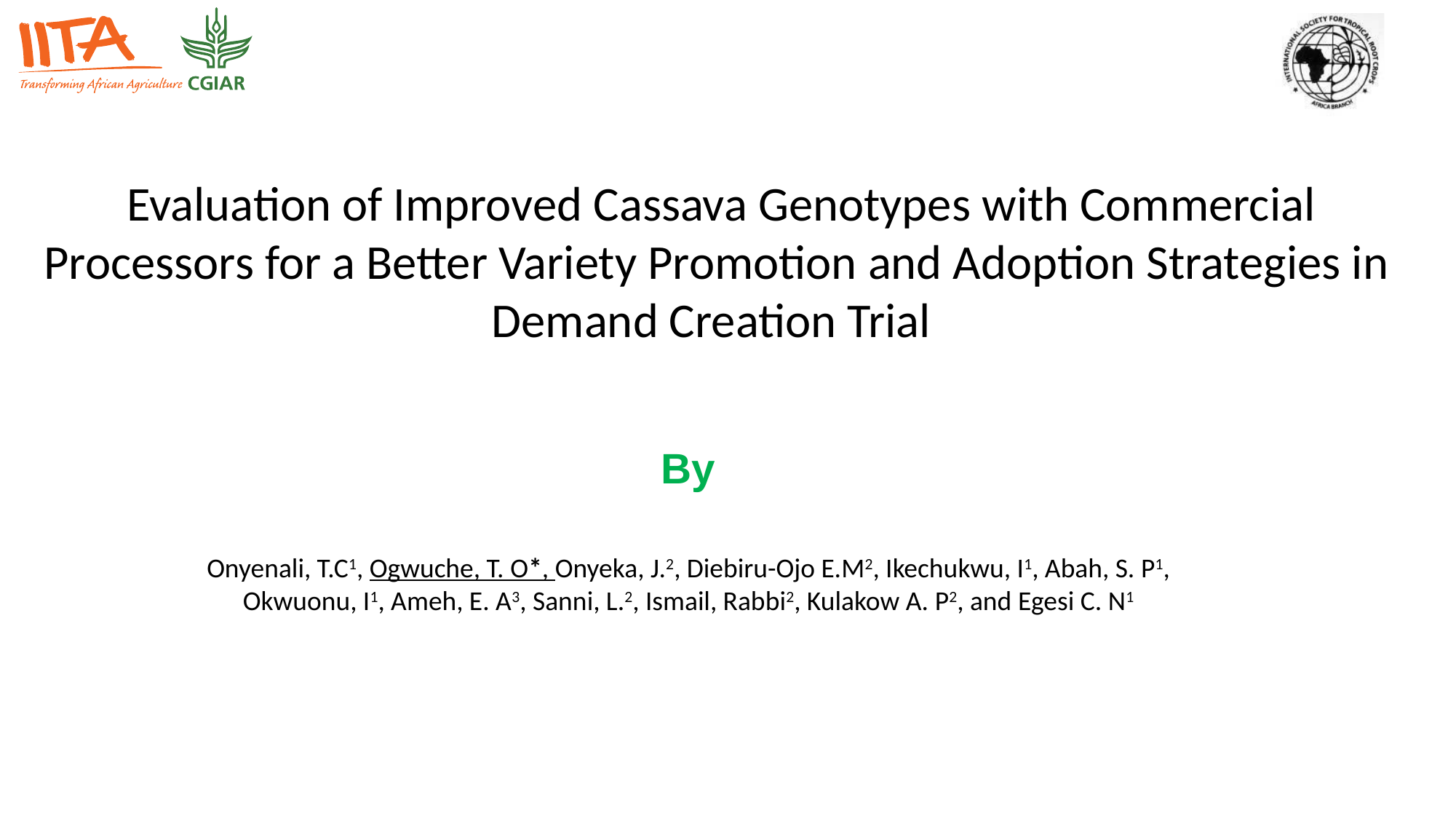

Evaluation of Improved Cassava Genotypes with Commercial Processors for a Better Variety Promotion and Adoption Strategies in Demand Creation Trial
By
Onyenali, T.C1, Ogwuche, T. O*, Onyeka, J.2, Diebiru-Ojo E.M2, Ikechukwu, I1, Abah, S. P1, Okwuonu, I1, Ameh, E. A3, Sanni, L.2, Ismail, Rabbi2, Kulakow A. P2, and Egesi C. N1

## Slide 2
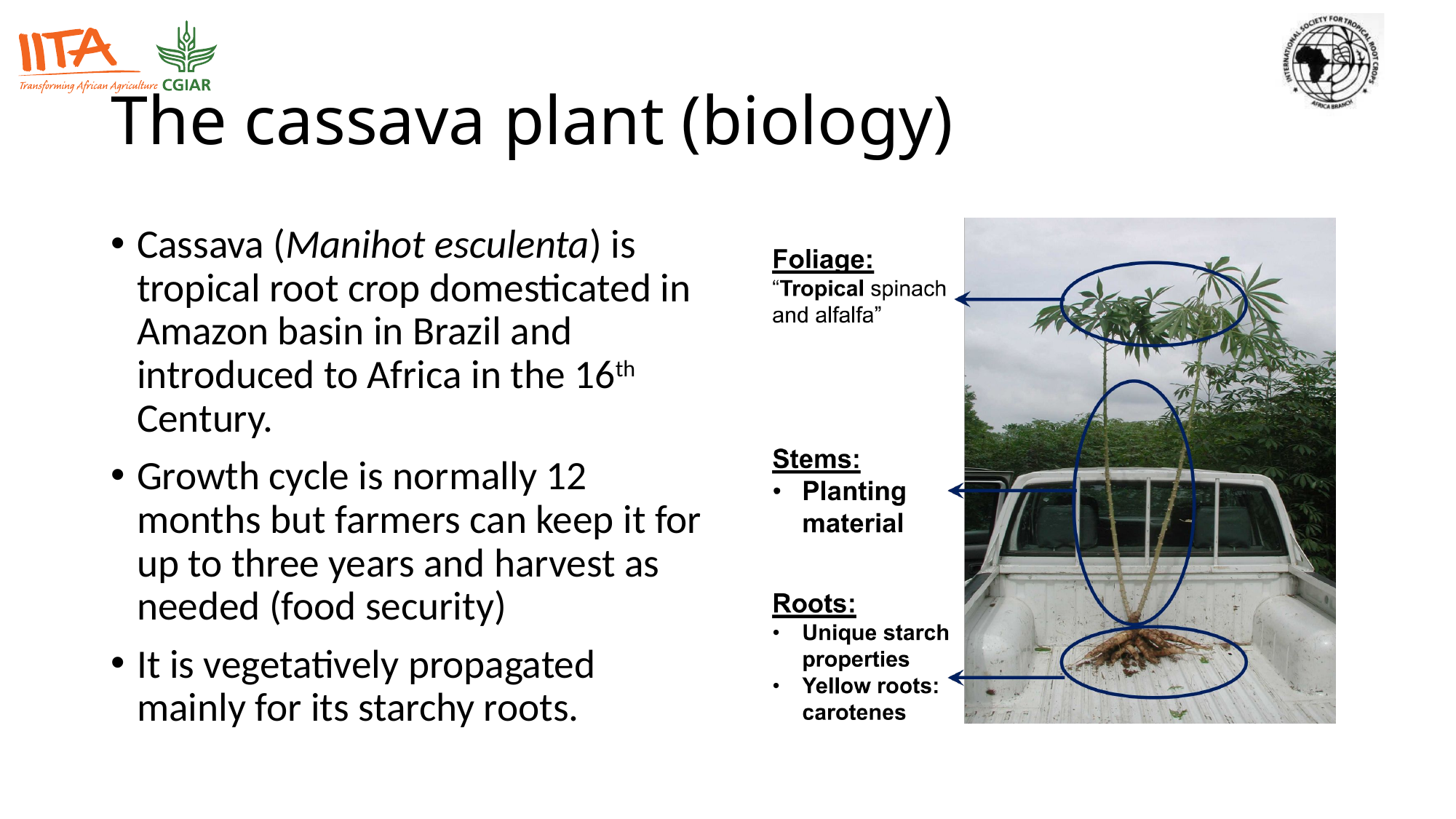

# The cassava plant (biology)
Cassava (Manihot esculenta) is tropical root crop domesticated in Amazon basin in Brazil and introduced to Africa in the 16th Century.
Growth cycle is normally 12 months but farmers can keep it for up to three years and harvest as needed (food security)
It is vegetatively propagated mainly for its starchy roots.

## Slide 3
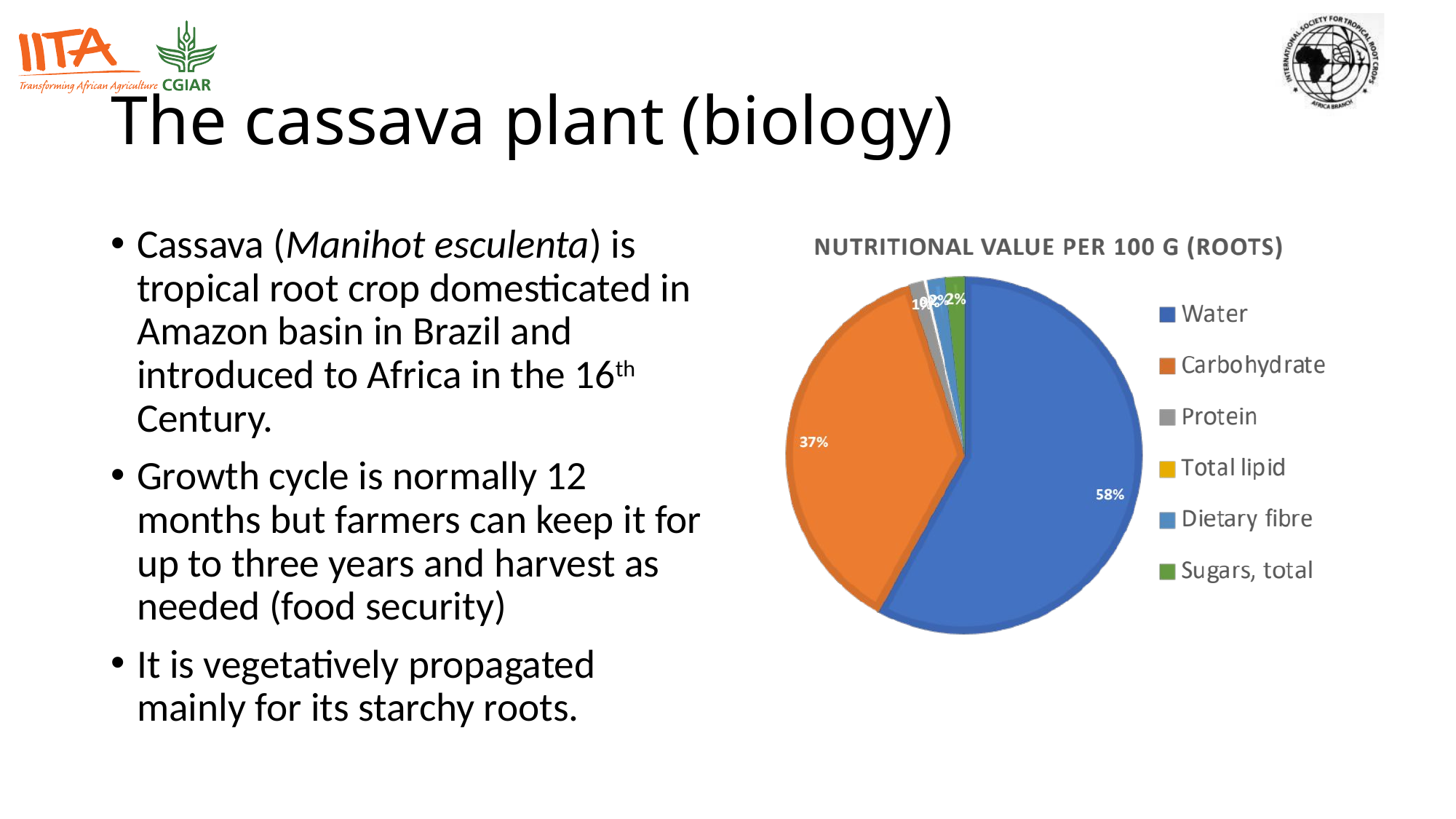

# The cassava plant (biology)
Cassava (Manihot esculenta) is tropical root crop domesticated in Amazon basin in Brazil and introduced to Africa in the 16th Century.
Growth cycle is normally 12 months but farmers can keep it for up to three years and harvest as needed (food security)
It is vegetatively propagated mainly for its starchy roots.

## Slide 4
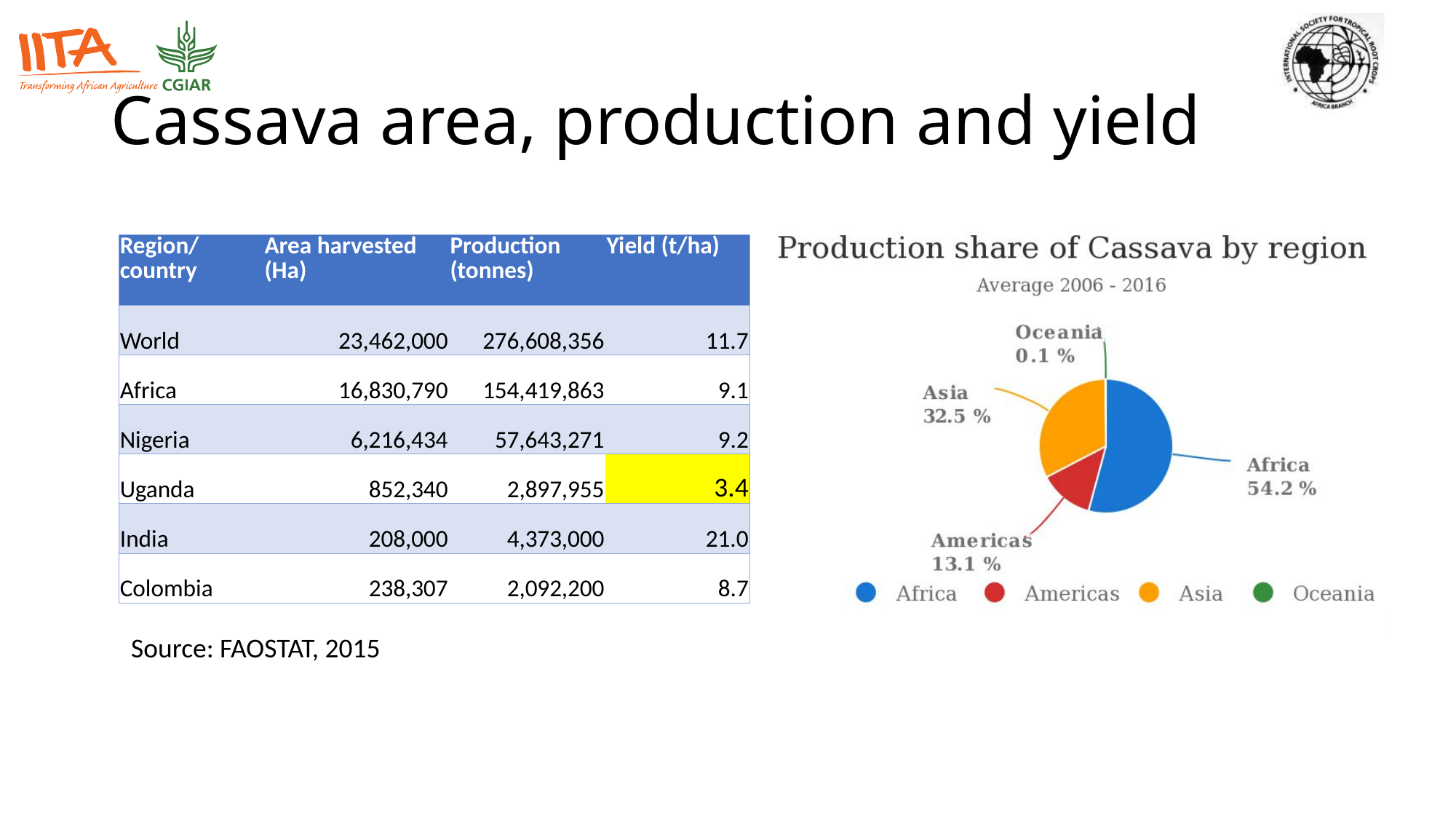

# Cassava area, production and yield
| Region/ country | Area harvested (Ha) | Production (tonnes) | Yield (t/ha) |
| --- | --- | --- | --- |
| World | 23,462,000 | 276,608,356 | 11.7 |
| Africa | 16,830,790 | 154,419,863 | 9.1 |
| Nigeria | 6,216,434 | 57,643,271 | 9.2 |
| Uganda | 852,340 | 2,897,955 | 3.4 |
| India | 208,000 | 4,373,000 | 21.0 |
| Colombia | 238,307 | 2,092,200 | 8.7 |
Source: FAOSTAT, 2015

## Slide 5
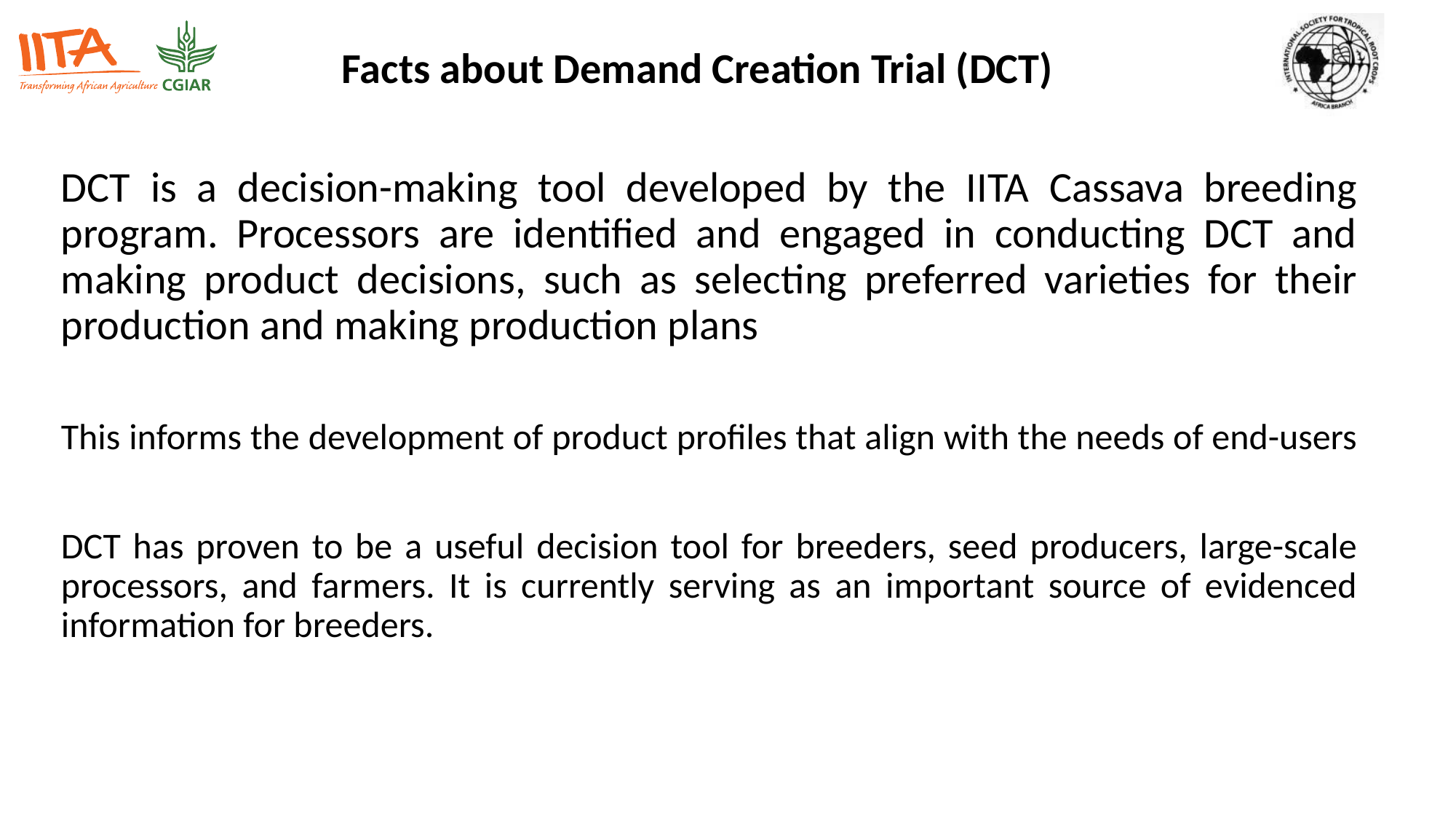

# Facts about Demand Creation Trial (DCT)
DCT is a decision-making tool developed by the IITA Cassava breeding program. Processors are identified and engaged in conducting DCT and making product decisions, such as selecting preferred varieties for their production and making production plans
This informs the development of product profiles that align with the needs of end-users
DCT has proven to be a useful decision tool for breeders, seed producers, large-scale processors, and farmers. It is currently serving as an important source of evidenced information for breeders.

## Slide 6
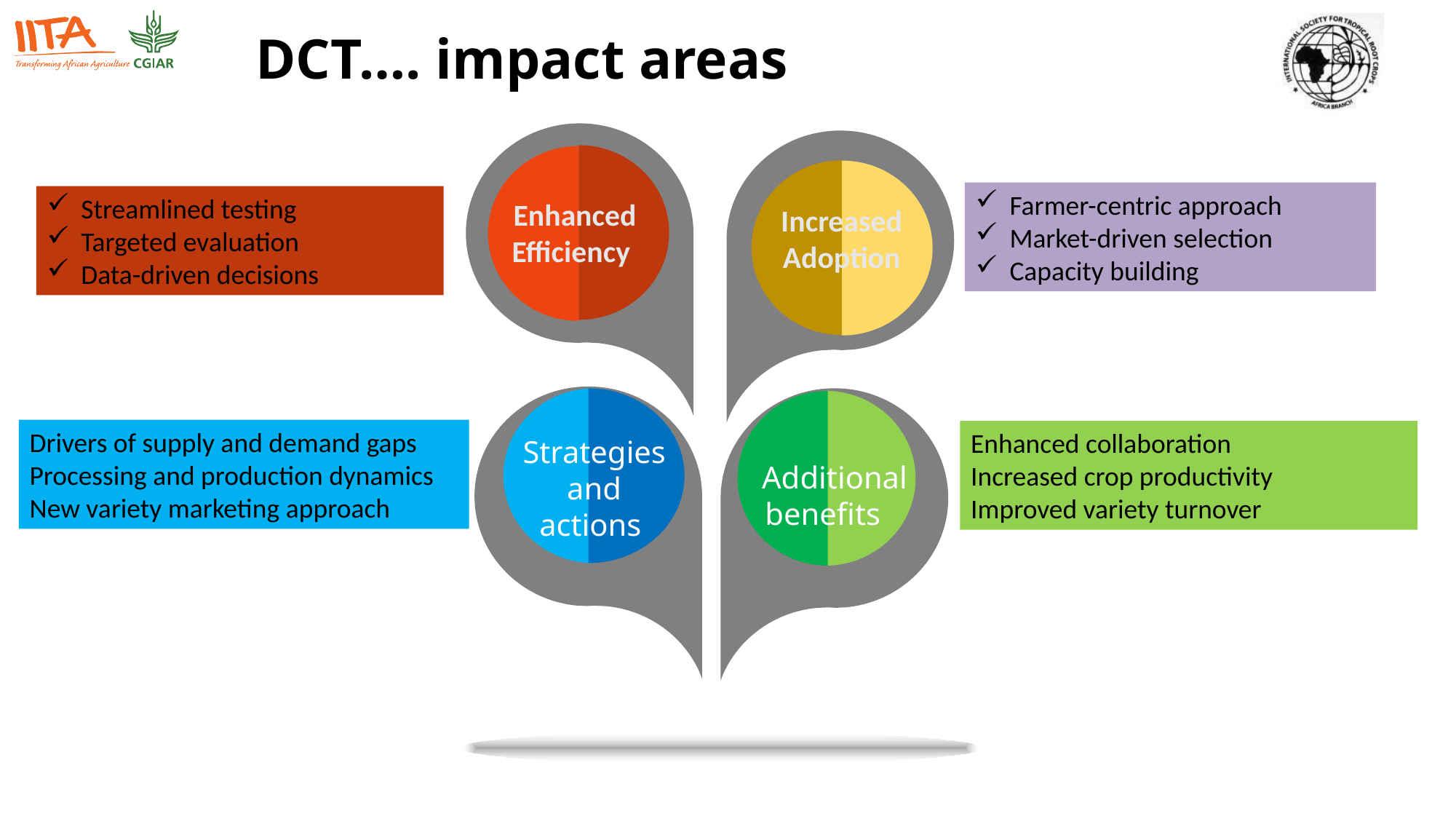

# DCT…. impact areas
Farmer-centric approach
Market-driven selection
Capacity building
Streamlined testing
Targeted evaluation
Data-driven decisions
Enhanced Efficiency
Increased Adoption
Drivers of supply and demand gaps
Processing and production dynamics
New variety marketing approach
Enhanced collaboration
Increased crop productivity
Improved variety turnover
Strategies and actions
Additional benefits

## Slide 7
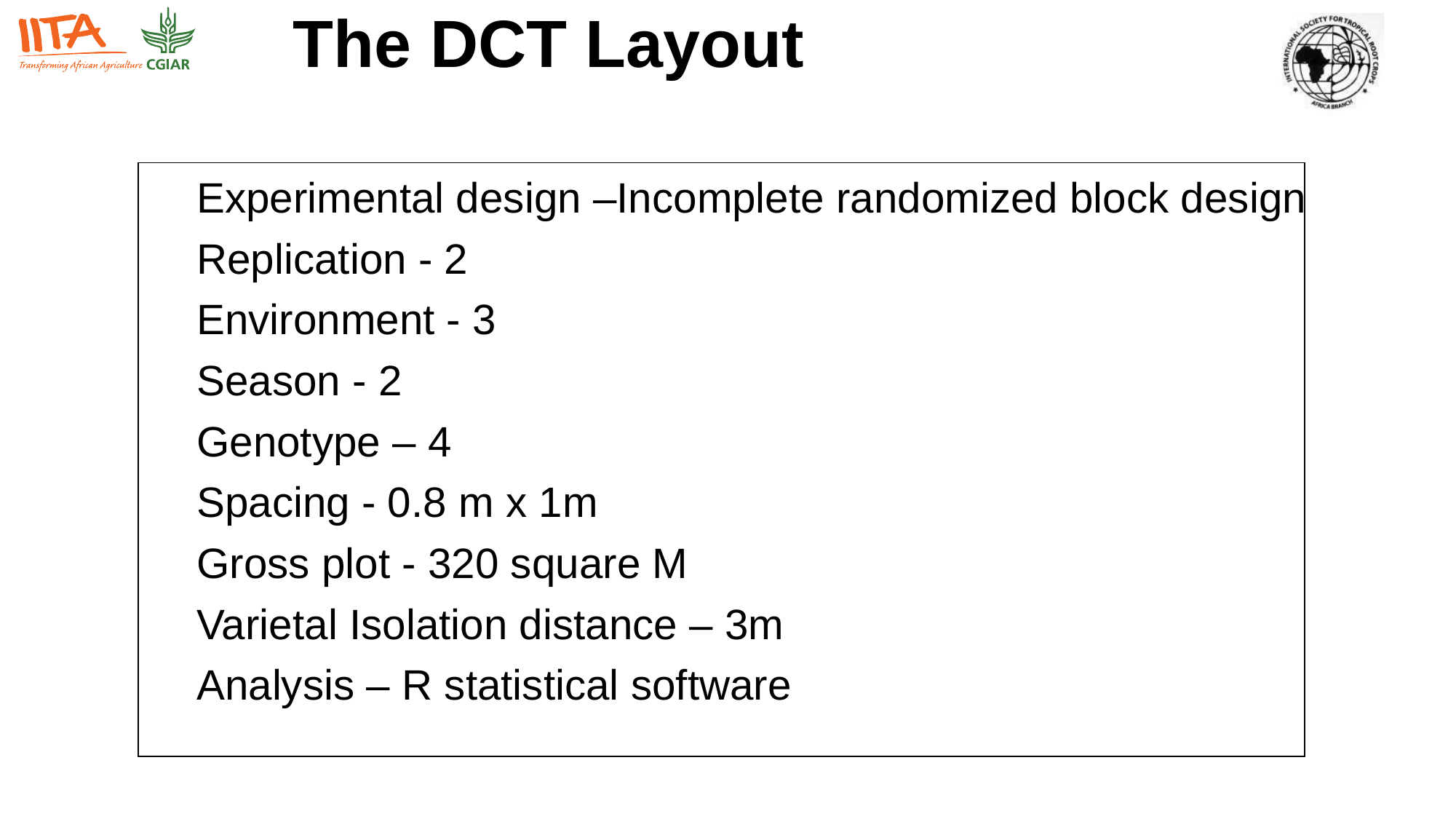

# The DCT Layout
| |
| --- |
Experimental design –Incomplete randomized block design
Replication - 2
Environment - 3
Season - 2
Genotype – 4
Spacing - 0.8 m x 1m
Gross plot - 320 square M
Varietal Isolation distance – 3m
Analysis – R statistical software

## Slide 8
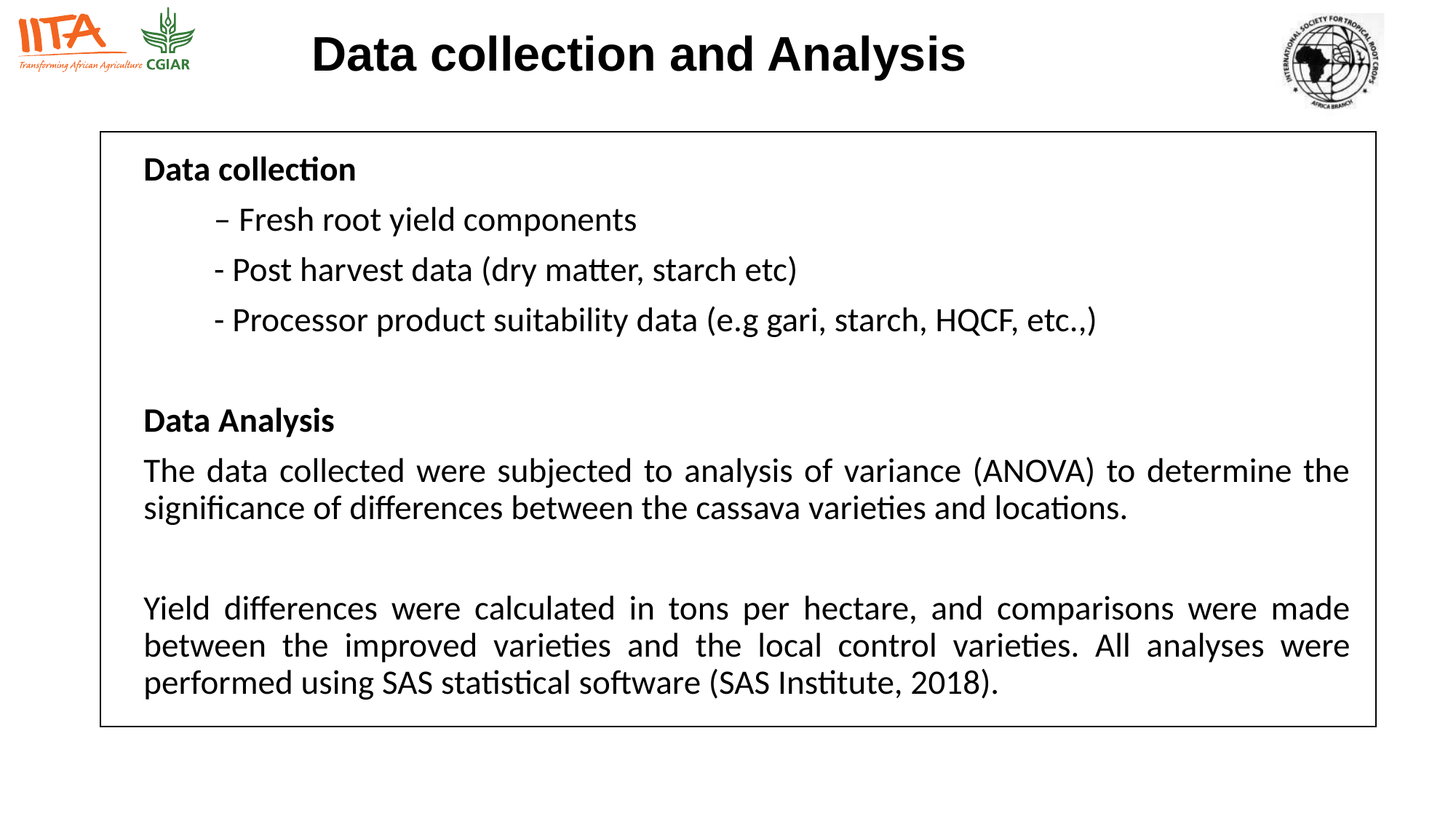

# Data collection and Analysis
| |
| --- |
Data collection
 – Fresh root yield components
 - Post harvest data (dry matter, starch etc)
 - Processor product suitability data (e.g gari, starch, HQCF, etc.,)
Data Analysis
The data collected were subjected to analysis of variance (ANOVA) to determine the significance of differences between the cassava varieties and locations.
Yield differences were calculated in tons per hectare, and comparisons were made between the improved varieties and the local control varieties. All analyses were performed using SAS statistical software (SAS Institute, 2018).

## Slide 9
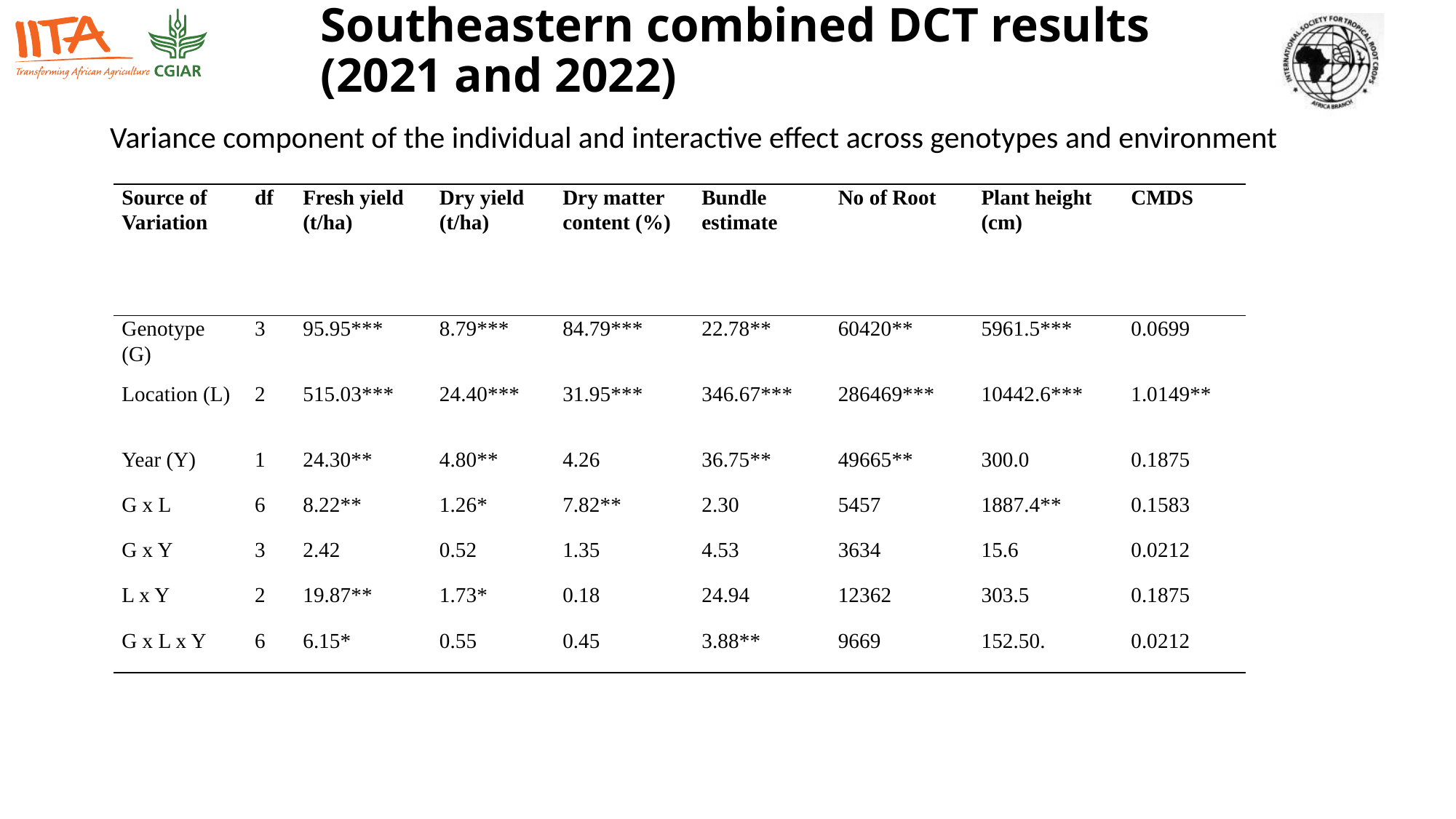

# Southeastern combined DCT results (2021 and 2022)
Variance component of the individual and interactive effect across genotypes and environment
| Source of Variation | df | Fresh yield (t/ha) | Dry yield (t/ha) | Dry matter content (%) | Bundle estimate | No of Root | Plant height (cm) | CMDS |
| --- | --- | --- | --- | --- | --- | --- | --- | --- |
| Genotype (G) | 3 | 95.95\*\*\* | 8.79\*\*\* | 84.79\*\*\* | 22.78\*\* | 60420\*\* | 5961.5\*\*\* | 0.0699 |
| Location (L) | 2 | 515.03\*\*\* | 24.40\*\*\* | 31.95\*\*\* | 346.67\*\*\* | 286469\*\*\* | 10442.6\*\*\* | 1.0149\*\* |
| Year (Y) | 1 | 24.30\*\* | 4.80\*\* | 4.26 | 36.75\*\* | 49665\*\* | 300.0 | 0.1875 |
| G x L | 6 | 8.22\*\* | 1.26\* | 7.82\*\* | 2.30 | 5457 | 1887.4\*\* | 0.1583 |
| G x Y | 3 | 2.42 | 0.52 | 1.35 | 4.53 | 3634 | 15.6 | 0.0212 |
| L x Y | 2 | 19.87\*\* | 1.73\* | 0.18 | 24.94 | 12362 | 303.5 | 0.1875 |
| G x L x Y | 6 | 6.15\* | 0.55 | 0.45 | 3.88\*\* | 9669 | 152.50. | 0.0212 |

## Slide 10
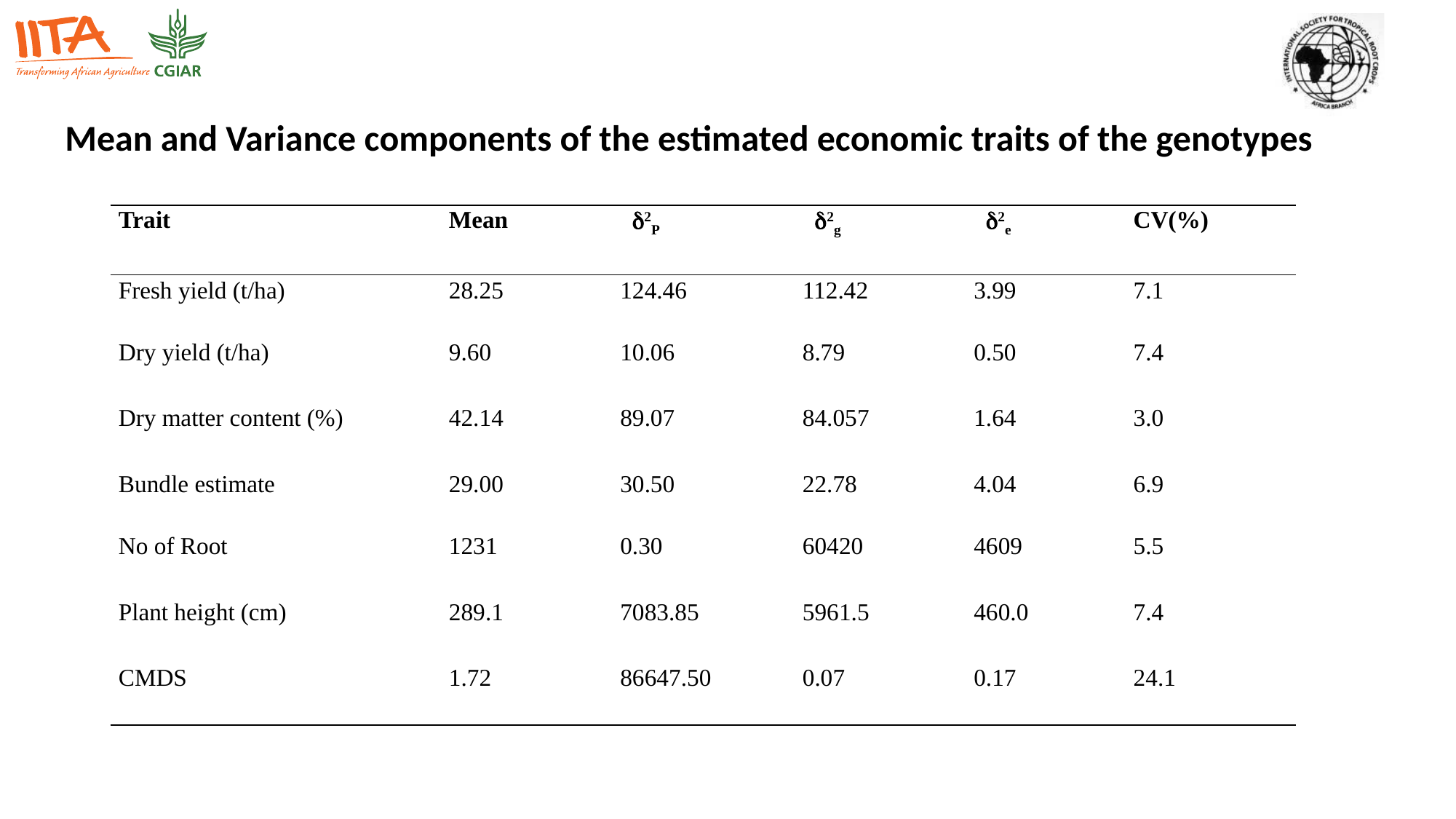

Mean and Variance components of the estimated economic traits of the genotypes
| Trait | Mean | 2P | 2g | 2e | CV(%) |
| --- | --- | --- | --- | --- | --- |
| Fresh yield (t/ha) | 28.25 | 124.46 | 112.42 | 3.99 | 7.1 |
| Dry yield (t/ha) | 9.60 | 10.06 | 8.79 | 0.50 | 7.4 |
| Dry matter content (%) | 42.14 | 89.07 | 84.057 | 1.64 | 3.0 |
| Bundle estimate | 29.00 | 30.50 | 22.78 | 4.04 | 6.9 |
| No of Root | 1231 | 0.30 | 60420 | 4609 | 5.5 |
| Plant height (cm) | 289.1 | 7083.85 | 5961.5 | 460.0 | 7.4 |
| CMDS | 1.72 | 86647.50 | 0.07 | 0.17 | 24.1 |

## Slide 11
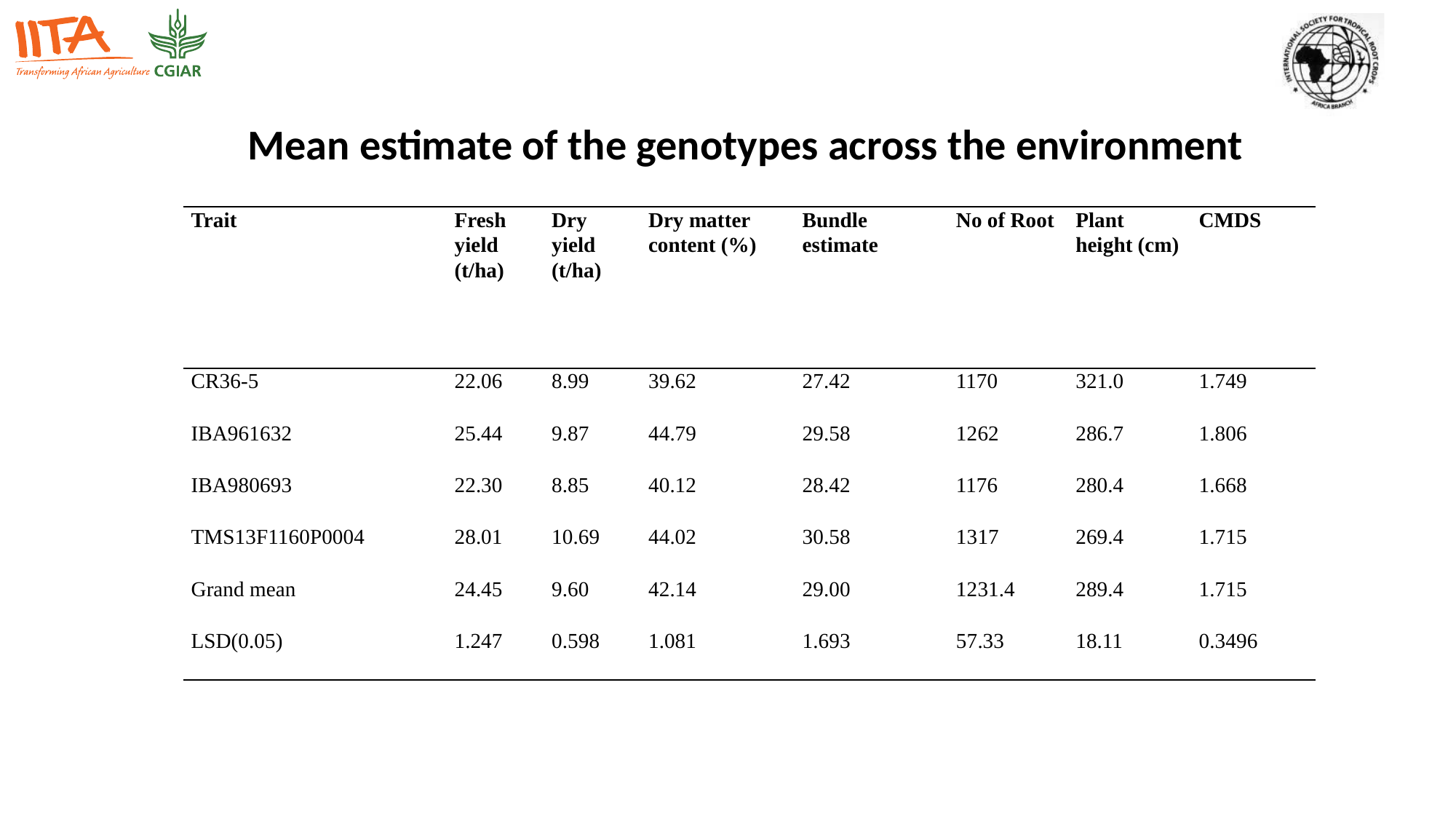

Mean estimate of the genotypes across the environment
| Trait | Fresh yield (t/ha) | Dry yield (t/ha) | Dry matter content (%) | Bundle estimate | No of Root | Plant height (cm) | CMDS |
| --- | --- | --- | --- | --- | --- | --- | --- |
| CR36-5 | 22.06 | 8.99 | 39.62 | 27.42 | 1170 | 321.0 | 1.749 |
| IBA961632 | 25.44 | 9.87 | 44.79 | 29.58 | 1262 | 286.7 | 1.806 |
| IBA980693 | 22.30 | 8.85 | 40.12 | 28.42 | 1176 | 280.4 | 1.668 |
| TMS13F1160P0004 | 28.01 | 10.69 | 44.02 | 30.58 | 1317 | 269.4 | 1.715 |
| Grand mean | 24.45 | 9.60 | 42.14 | 29.00 | 1231.4 | 289.4 | 1.715 |
| LSD(0.05) | 1.247 | 0.598 | 1.081 | 1.693 | 57.33 | 18.11 | 0.3496 |

## Slide 12
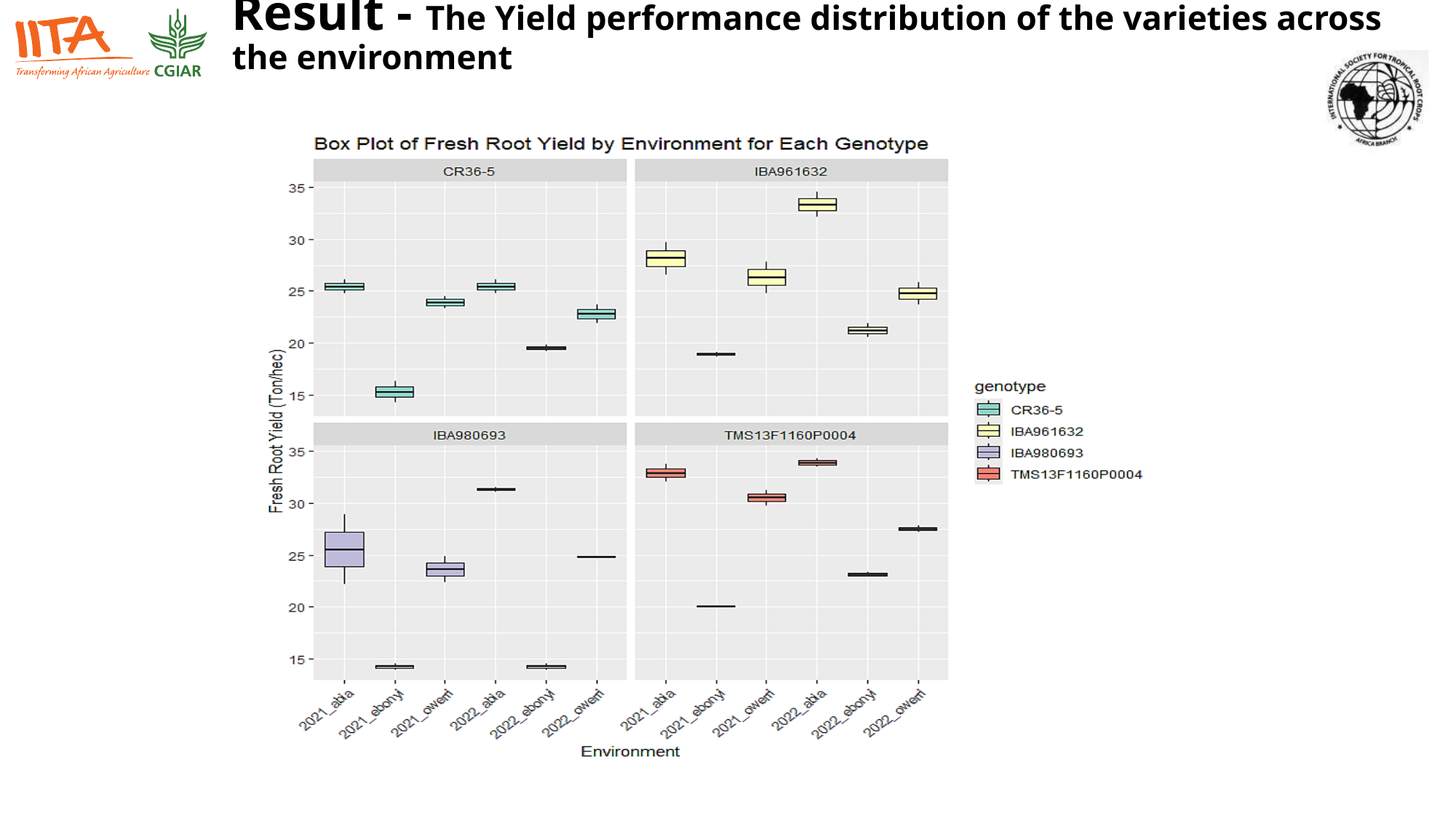

# Result - The Yield performance distribution of the varieties across the environment

## Slide 13
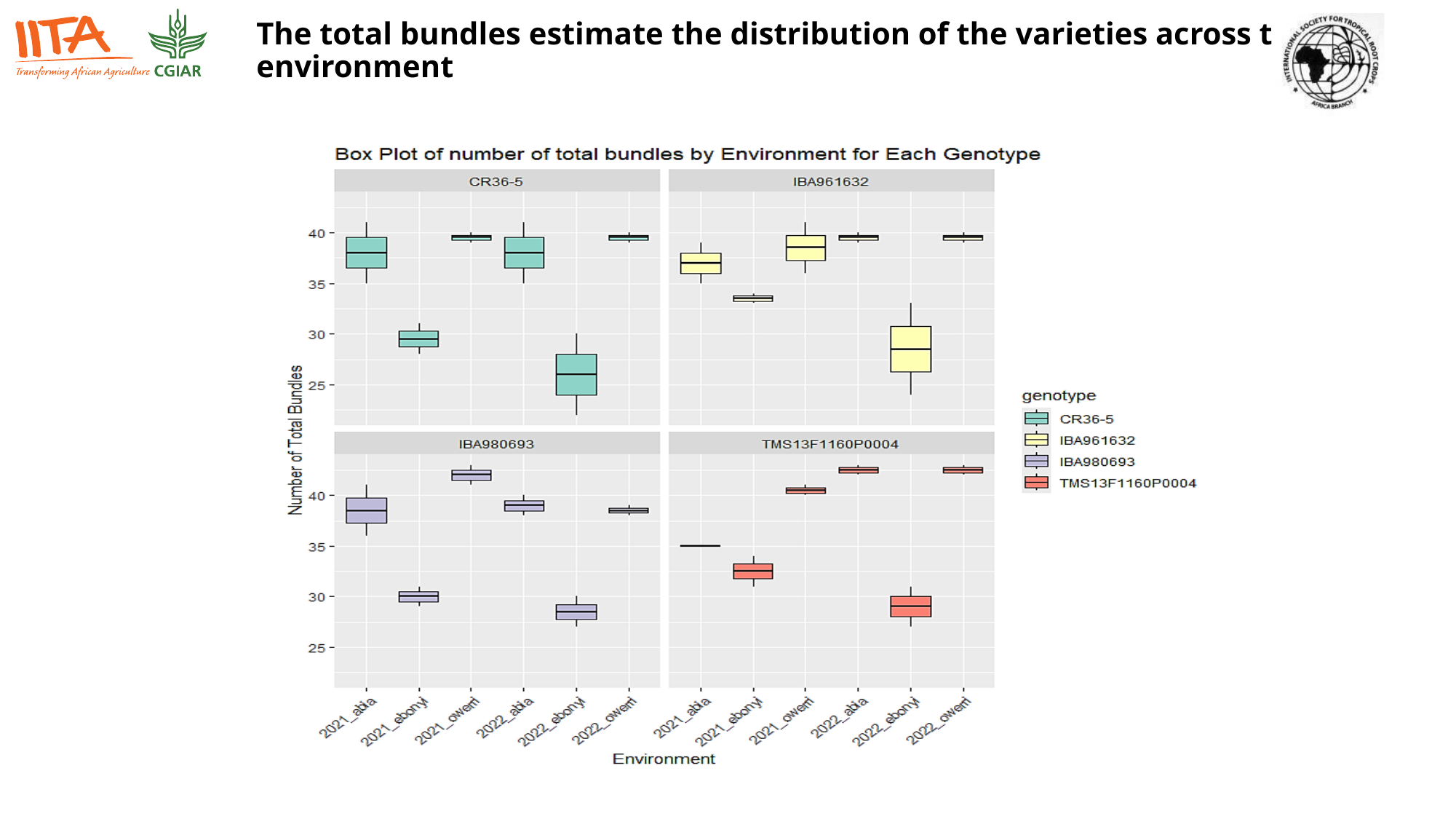

# The total bundles estimate the distribution of the varieties across the environment

## Slide 14
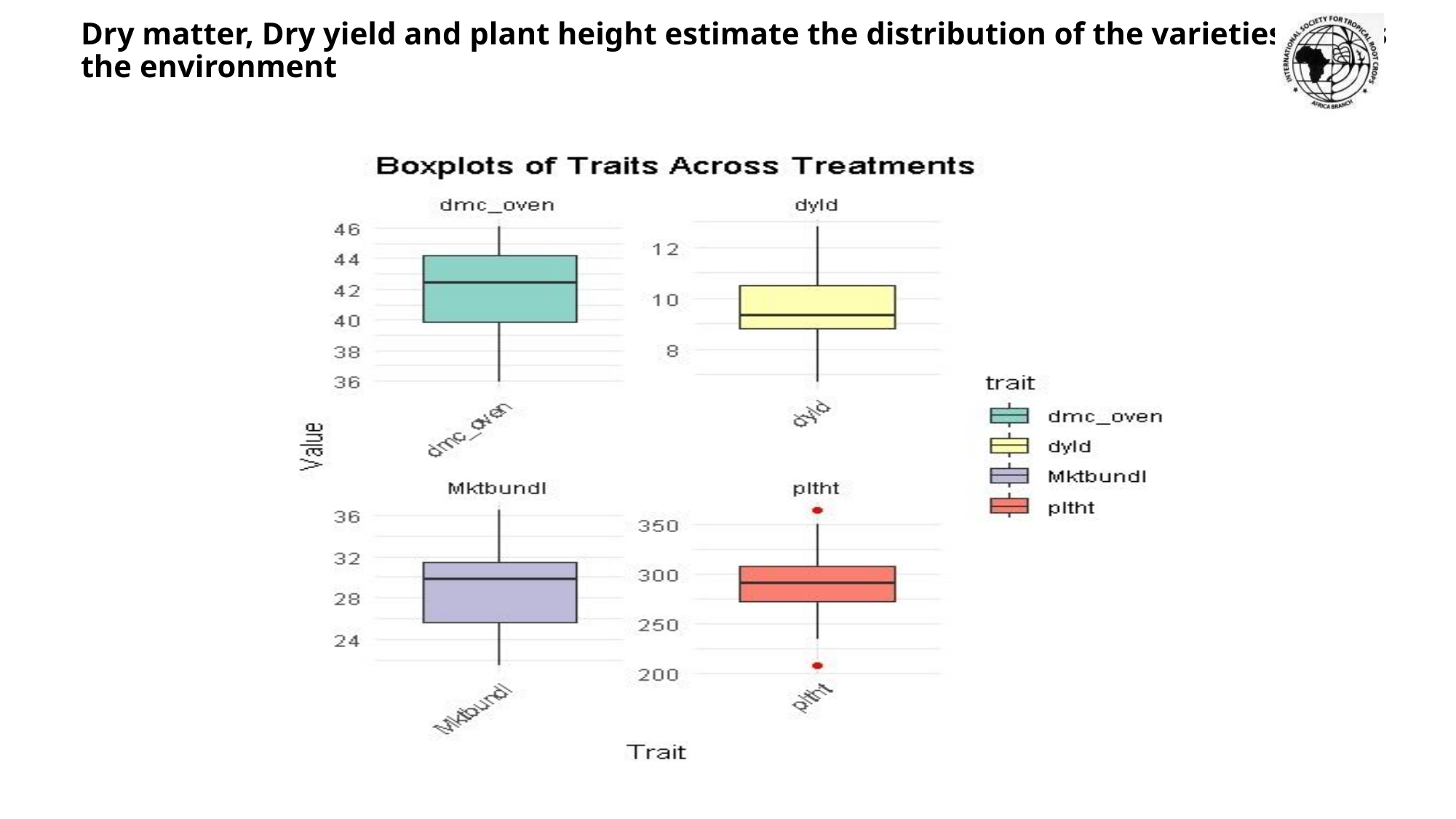

# Dry matter, Dry yield and plant height estimate the distribution of the varieties across the environment

## Slide 15
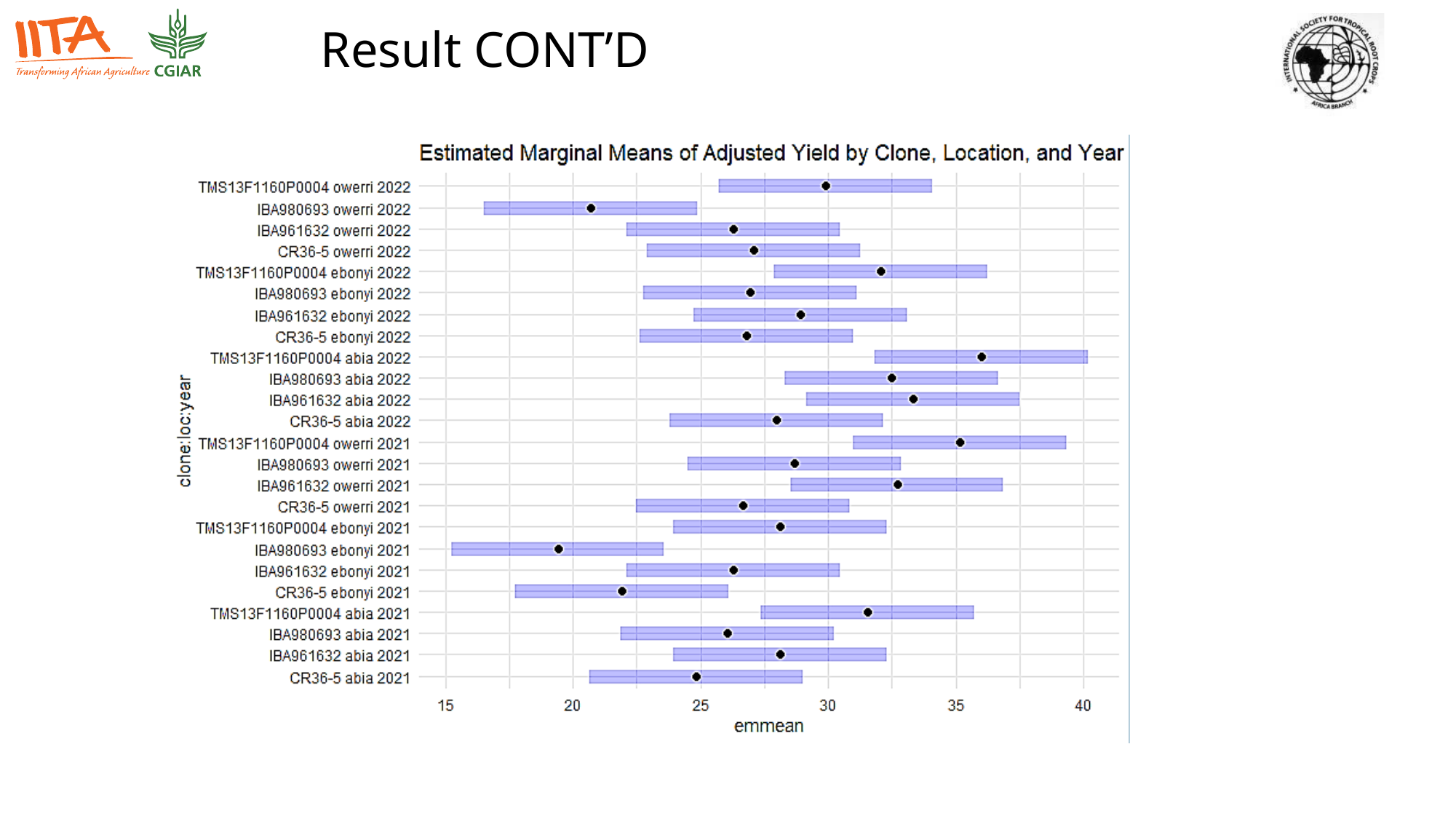

# Result CONT’D

## Slide 16
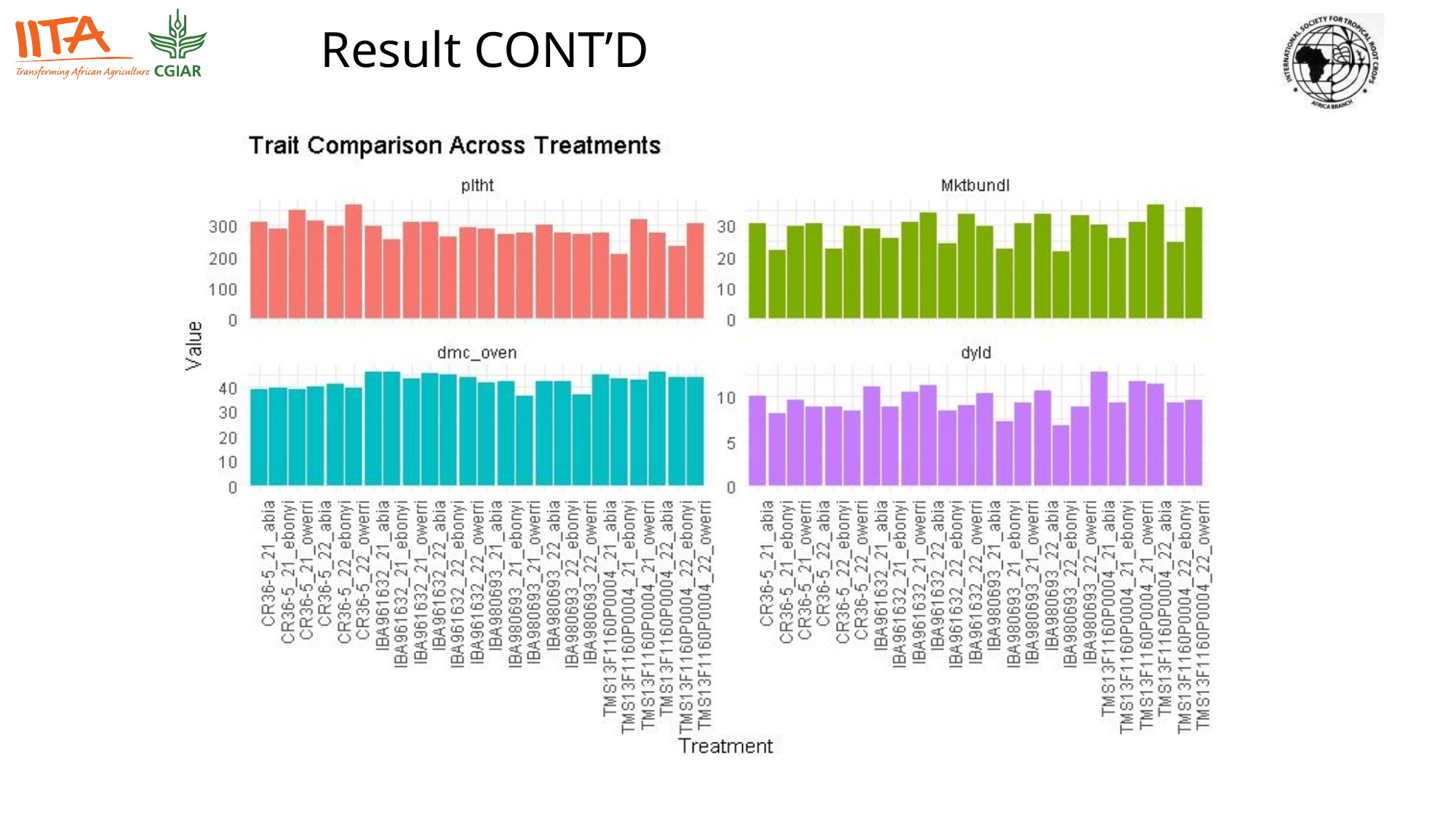

# Result CONT’D

## Slide 17
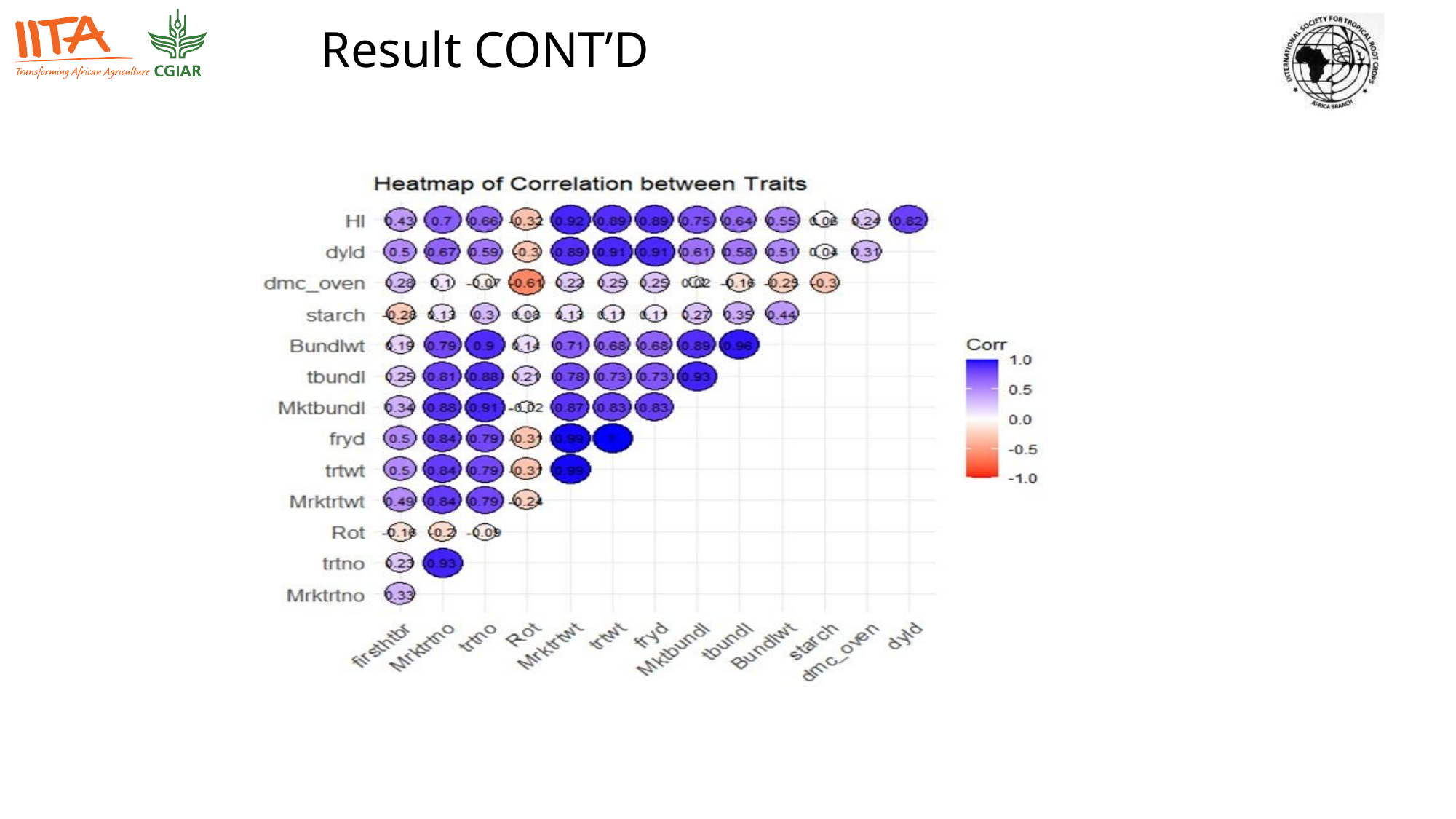

# Result CONT’D

## Slide 18
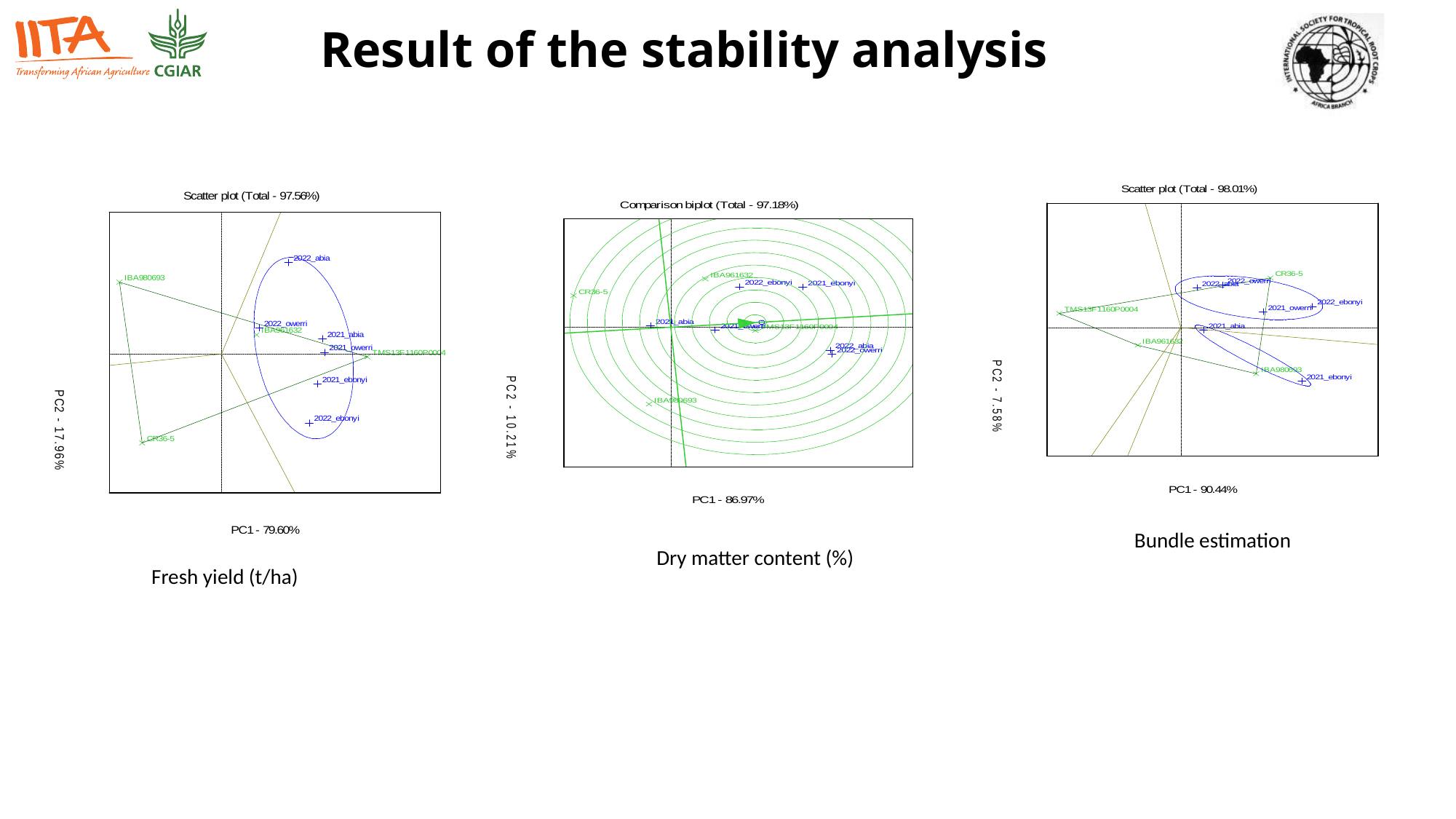

# Result of the stability analysis
Bundle estimation
Dry matter content (%)
Fresh yield (t/ha)

## Slide 19
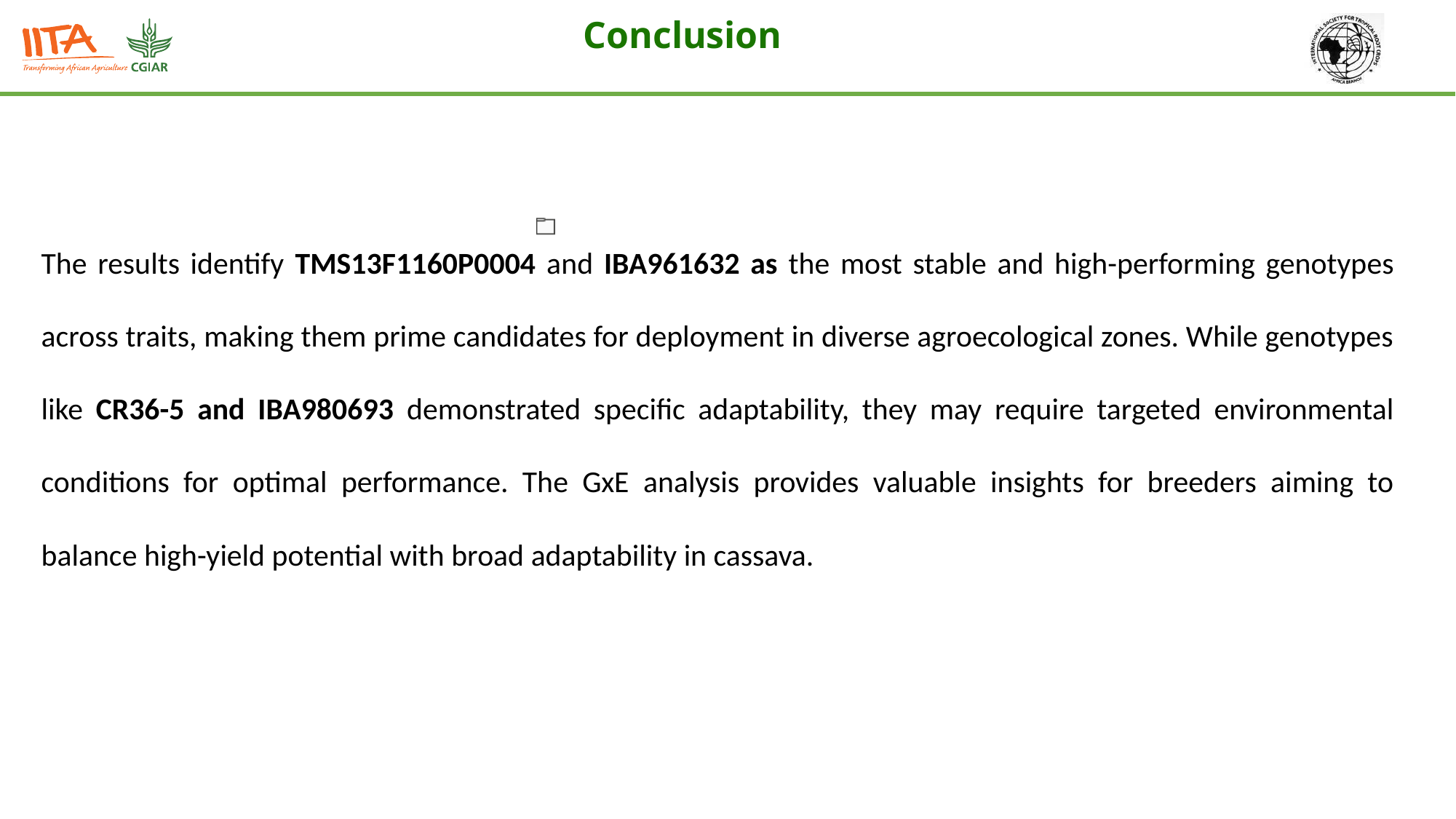

Conclusion
The results identify TMS13F1160P0004 and IBA961632 as the most stable and high-performing genotypes across traits, making them prime candidates for deployment in diverse agroecological zones. While genotypes like CR36-5 and IBA980693 demonstrated specific adaptability, they may require targeted environmental conditions for optimal performance. The GxE analysis provides valuable insights for breeders aiming to balance high-yield potential with broad adaptability in cassava.

## Slide 20
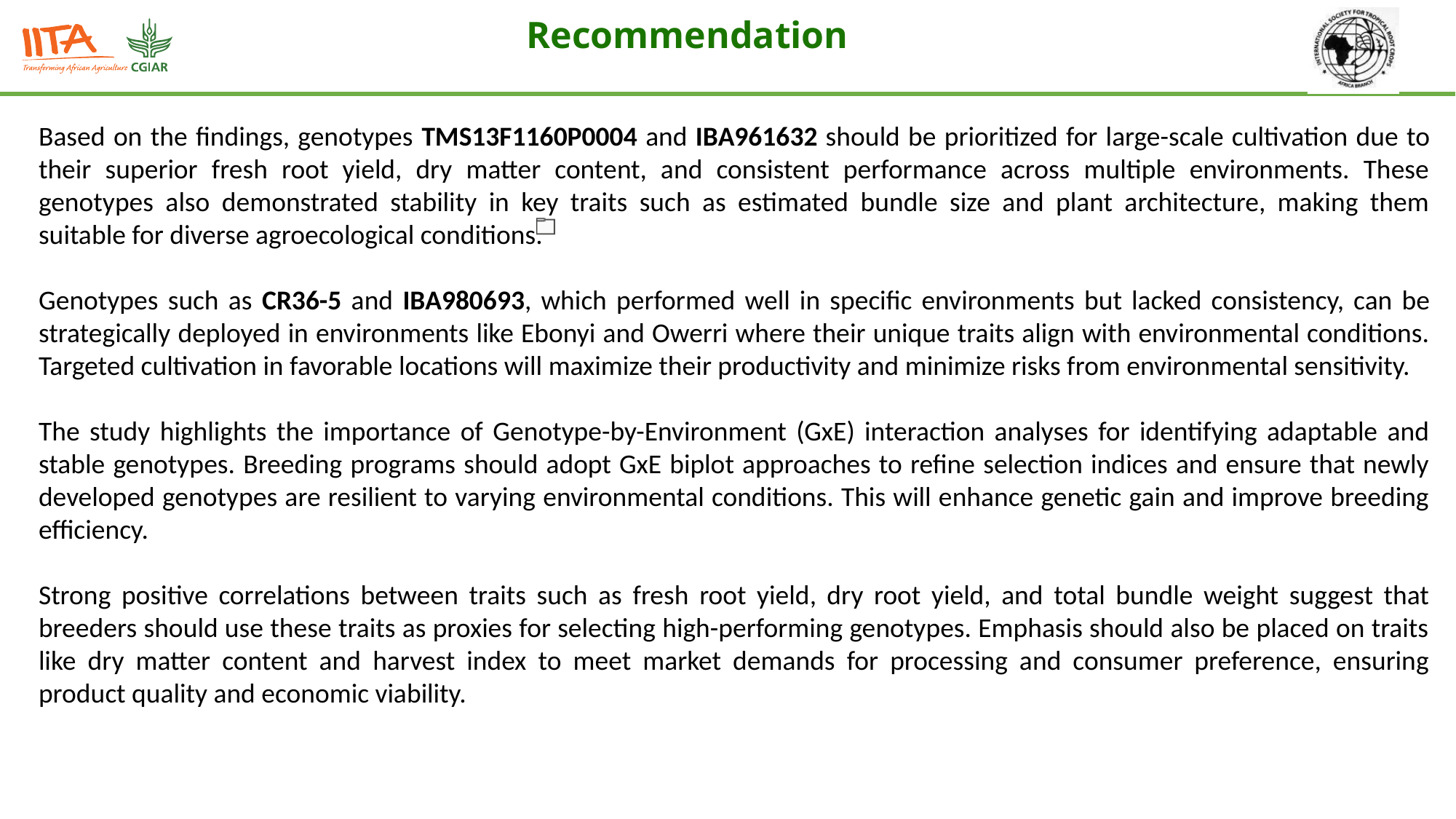

Recommendation
Based on the findings, genotypes TMS13F1160P0004 and IBA961632 should be prioritized for large-scale cultivation due to their superior fresh root yield, dry matter content, and consistent performance across multiple environments. These genotypes also demonstrated stability in key traits such as estimated bundle size and plant architecture, making them suitable for diverse agroecological conditions.
Genotypes such as CR36-5 and IBA980693, which performed well in specific environments but lacked consistency, can be strategically deployed in environments like Ebonyi and Owerri where their unique traits align with environmental conditions. Targeted cultivation in favorable locations will maximize their productivity and minimize risks from environmental sensitivity.
The study highlights the importance of Genotype-by-Environment (GxE) interaction analyses for identifying adaptable and stable genotypes. Breeding programs should adopt GxE biplot approaches to refine selection indices and ensure that newly developed genotypes are resilient to varying environmental conditions. This will enhance genetic gain and improve breeding efficiency.
Strong positive correlations between traits such as fresh root yield, dry root yield, and total bundle weight suggest that breeders should use these traits as proxies for selecting high-performing genotypes. Emphasis should also be placed on traits like dry matter content and harvest index to meet market demands for processing and consumer preference, ensuring product quality and economic viability.

## Slide 21
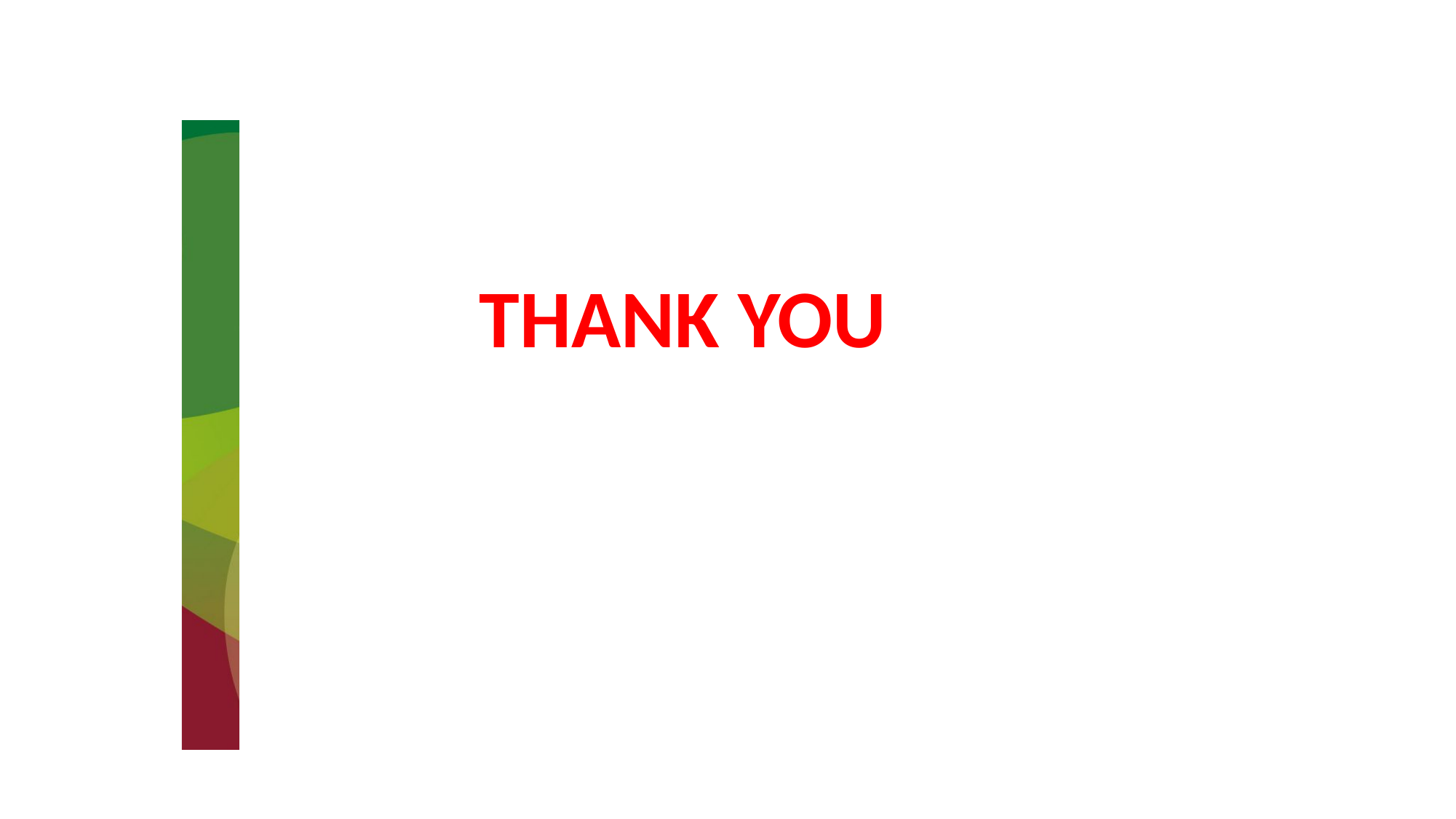

THANK YOU
